# In silico analysis of fungal small RNA accumulation reveals putative plant mRNA targets in the symbiosis between an arbuscular mycorrhizal fungus and its host plant

**DOI:** 10.1186/s12864-019-5561-0

**Published:** 2019-03-04

**Authors:** Alessandro Silvestri, Valentina Fiorilli, Laura Miozzi, Gian Paolo Accotto, Massimo Turina, Luisa Lanfranco

**Affiliations:** 1 Department of Life Sciences and Systems Biology, University of Torino, Viale P.A. Mattioli 25, 10125 Torino, Italy; 2 Institute for Sustainable Plant Protection – CNR Torino, Strada delle Cacce 73, 10131 Torino, Italy

**Keywords:** Arbuscular mycorrhizal fungi, *Rhizophagus*, Small RNAs, microRNA-like, RNA interference

## Abstract

**Background:**

Small RNAs (sRNAs) are short non-coding RNA molecules (20–30 nt) that regulate gene expression at transcriptional or post-transcriptional levels in many eukaryotic organisms, through a mechanism known as RNA interference (RNAi). Recent studies have highlighted that they are also involved in cross-kingdom communication: sRNAs can move across the contact surfaces from “donor” to “receiver” organisms and, once in the host cells of the receiver, they can target specific mRNAs, leading to a modulation of host metabolic pathways and defense responses. Very little is known about RNAi mechanism and sRNAs occurrence in Arbuscular Mycorrhizal Fungi (AMF), an important component of the plant root microbiota that provide several benefits to host plants, such as improved mineral uptake and tolerance to biotic and abiotic stress.

**Results:**

Taking advantage of the available genomic resources for the AMF *Rhizophagus irregularis* we described its putative RNAi machinery, which is characterized by a single *Dicer*-like (*DCL*) gene and an unusual expansion of *Argonaute*-like (*AGO*-like) and *RNA-dependent RNA polymerase* (*RdRp*) gene families. In silico investigations of previously published transcriptomic data and experimental assays carried out in this work provided evidence of gene expression for most of the identified sequences. Focusing on the symbiosis between *R. irregularis* and the model plant *Medicago truncatula*, we characterized the fungal sRNA population, highlighting the occurrence of an active sRNA-generating pathway and the presence of microRNA-like sequences. In silico analyses, supported by host plant degradome data, revealed that several fungal sRNAs have the potential to target *M. truncatula* transcripts, including some specific mRNA already shown to be modulated in roots upon AMF colonization.

**Conclusions:**

The identification of RNAi-related genes, together with the characterization of the sRNAs population, suggest that *R. irregularis* is equipped with a functional sRNA-generating pathway. Moreover, the in silico analysis predicted 237 plant transcripts as putative targets of specific fungal sRNAs suggesting that cross-kingdom post-transcriptional gene silencing may occur during AMF colonization.

**Electronic supplementary material:**

The online version of this article (10.1186/s12864-019-5561-0) contains supplementary material, which is available to authorized users.

## Background

*Rhizophagus irregularis* is a model system for arbuscular mycorrhizal fungi (AMF); it belongs to Glomeromycotina [1], a group of soil fungi able to form a mutualistic symbiosis with the majority of land plants. AMF fungi facilitate the supply of water and nutrients to host plants in return of fixed carbon [2]. However, the beneficial effects of the AM symbiosis go beyond an improved mineral nutrition and includes enhanced tolerance to biotic and abiotic stress [3].

A long history of co-evolution characterizes this unique plant-fungus association where the typical highly branched fungal structures (arbuscules), which develop inside cortical cells, represent a clear sign of the occurrence of fine-tuned regulatory circuits in both partners. Such an intimate colonization of plant tissues relies on an efficient molecular communication system, which occurs before the contact, and on extensive structural and metabolic rearrangements on both plant and fungal sides, which have been only partially described [2, 4]. Transcriptomic studies, mainly focused on plant protein-encoding genes, have been instrumental to describe the molecular reprogramming that the AMF colonization induces in different host plants not only locally (roots; [5–7]) but also systemically (shoot and fruit; [8, 9]) level. Nevertheless, investigations on transcript profiles have been performed to a lower extent also on the AMF [10].

Regulation of gene expression relies on several factors related to transcriptional, post-transcriptional and translational events. The most recently characterized level of regulation relies on the RNA interference (RNAi) mechanism and involves small RNAs (sRNAs): they are short non coding RNA molecules (20–30 nt) that can act at transcriptional or post-transcriptional level in many eukaryotic organisms [11, 12]. Basic enzymatic components of the RNAi response are an RNAse III protein, Dicer, that produces sRNAs from double-stranded RNAs (dsRNAs) and an Argonaute (AGO) protein, that uses these sRNAs to guide the selective and sequence-specific degradation, translational inhibition or transcriptional repression of the target [13]. An RNA-dependent RNA polymerase (RdRp) is also used in some organisms (nematodes, fungi and plants) to generate dsRNAs from aberrant RNAs and to amplify the silencing signal [13].

The main function initially ascribed to RNAi was the protection of the genome against transposons and exogenous sequences such as invading viruses or transgenes [12]. Later, it became clear that RNAi is also involved in the production of a variety of endogenous sRNAs, which participate, through the control of gene expression, in the regulation of several endogenous biological functions through the control of gene expression [12].

In the 1990s pioneering studies on the filamentous fungus *Neurospora crassa* were seminal to describe the phenomenon of RNAi in fungi [14]. Since then, investigations on RNAi components and sRNAs populations have been carried out on other fungi and indicated that many of them possess functional sRNAs while some species, such as *Saccharomyces cerevisiae* and *Ustilago maydis*, lost their RNAi capability [15, 16]. Furthermore, studies on fungal models and plant pathogens have shown that fungi may possess different classes of sRNAs, which are produced by multiple *Dicer*-dependent and *Dicer*-independent RNAi pathways [13]. Fungi are thus emerging fascinating systems to study RNAi-related processes and, because of their key position in the eukaryotic tree of life, they could provide insights on the evolution and diversification of RNAi.

Interestingly, recent investigations have highlighted that sRNAs are also involved in cross-kingdom communication [17–25]. In particular, concerning the interactions between plants-fungal pathogens or plants-parasitic plants, sRNAs can move across the contact surface, from “donor” to “receiver” organisms. Once in the host cells, sRNAs can target specific host mRNAs, sometimes triggering secondary sRNA production and thus leading to a modulation of host metabolic pathways and defense responses [26–28]. In case of parasitic/pathogenic organisms these findings are of great interest in light of the development of innovative crop defense strategies [23, 24, 29].

Currently very little is known about AMF RNAi machinery [30] and whether AMF possess a population of functional sRNAs. Furthermore, nothing is known about possible sRNAs trafficking and reciprocal sRNA-mediated communication between AMF and host plants. HIGS (host-induced gene silencing) and VIGS (virus induced gene silencing) have been shown to be successful tools for gene silencing in AMF [31–34] suggesting that RNA movement from the host to the fungus indeed occurs and RNAi-related mechanisms are active in AMF. In addition, it has been recently reported that several plant microRNAs are differentially expressed during the AM symbiosis [35–39]; although their functional roles remain widely unclear, some of them could represent potential candidate mobile sRNAs.

Aim of this work was to characterize the essential components of the RNA-mediated gene silencing machinery in the AMF *R. irregularis,* taking advantage of a newly published genome assembly [40]), and to characterize the population of *R. irregularis* sRNAs from extraradical mycelium and symbiotic tissues. We demonstrated that *R. irregularis* possesses key components of the RNAi machinery characterized by an unusual expansion of *AGO*-like (*Argonaute*-like) and *RdRp* gene families; furthermore, AMF sRNAs share structural properties with previously analyzed fungal sRNA datasets, including microRNA-like sequences. Finally, we identified in silico a list of predicted fungal sRNA-plant host mRNA target pairs possibly involved in cross-kingdom post-transcriptional gene silencing (PTGS) regulation during AMF colonization.

## Results

### RNAi machinery in *R. irregularis*

A survey of recently published genomic resources of the AMF *R. irregularis* [40] was performed to identify proteins belonging to the core eukaryotic RNAi machinery: Dicer-like (DCL), AGO and RdRp [13]. By keywords searches on JGI MycCosm portal [41], we found 1 DCL, 40 AGO-like and 21 RdRp putative homologous proteins that responded to the following criteria: the presence of two RNAse III domains for DCL [42], the presence of a piwi domain for AGO-like proteins, the presence of an RdRp domain for the RdRp [43]. A blastp search on the predicted *R. irregularis* proteome, using characterized DCL, AGO and RdRp from other fungi (the closely related *Mucor circinelloides* and the RNAi model systems *Neurospora crassa* and *Cryphonectria parasitica*) as queries, resulted in the same number of sequences obtained by keywords searches.

**Fig. 1:**
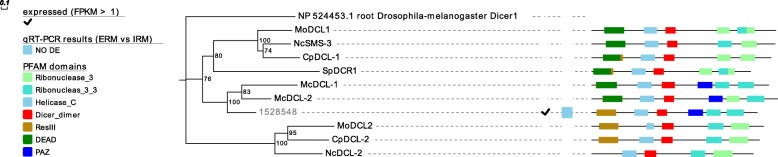
Summary of characterization of *R. irregularis* DCL including results of in silico gene expression, qRT-PCR, protein domains and phylogenetic analyses. Proteins are discernible by species according to a two-letter prefix: Mo = *Magnaporthe oryzae*,Nc = *Neurospora crassa*, Mc = *Mucor circinelloides*, Sp = *Schizosaccharomyces pombe*, Cp = *Cryphonectria parasitica*. The *Rhizophagus irregularis* protein is identified by JGI numeric code. Protein ID (NCBI): MoMDL1 = XP_003714515.1, MoMDL2 = XP_003715365.1, NcSMS-3 = XP_961898.1, NcDCL-2 = XP_963538.3, SpDCR1 = NP_588215.2, McDCL-1 = CAK32533.1, McDCL-2 = CAZ65730.1, CpDCL-1 = ABB00356.1, CpDCL-2 = ABB00357.1. The numbers at the nodes are bootstrap values (%) for 1000 replicates. ERM = extra radical mycelium, IRM = intra radical mycelium. Tree was rooted using *Drosophila melanogster* Dicer 1 (NCBI Reference Sequence: NP_524453.1). Figure was generated with Evolview v2

To further characterize the identified sequences, phylogenetic analyses were carried out. A first analysis, performed on DCL proteins, revealed that the only DCL of *R. irregularis* (1528548) is closely related to the two DCL described in *M. circinelloides* [44], consistent with the evolutionary relationships of the two taxonomic groups [1] (Fig. 1) and confirming the analysis carried out by Lee et al. (2018) on a previous, more fragmented, version of *R. irregularis* genome assembly [30]. Interestingly, Lee et al. (2018) also identified two additional prokaryotic (class I) ribonuclease III protein coding genes, which seem to derive from horizontal gene transfer from cyanobacteria [30].

**Fig. 2:**
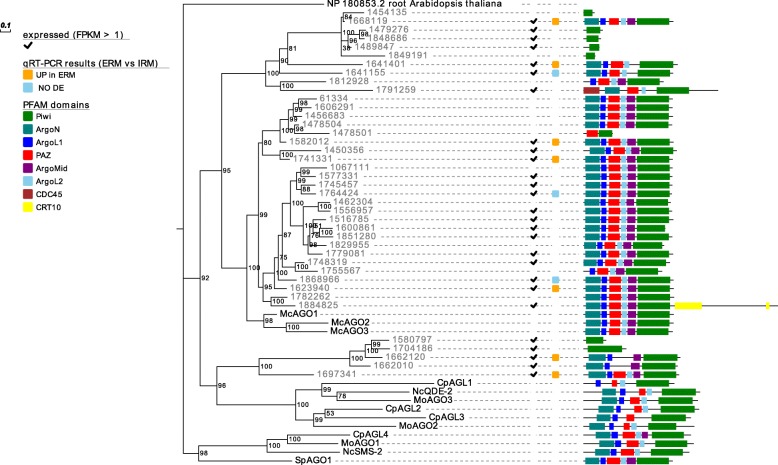
Summary of characterization of *Rhizophagus irregularis* AGO-like proteins including results of in silico gene expression, qRT-PCR, protein domains and phylogenetic analyses. Proteins are discernible by species according to a two-letter prefix: Mo = *Magnaporthe oryzae*,Nc = *Neurospora crassa*,Mc = *Mucor circinelloides*, Sp = *Schizosaccharomyces pombe*, Cp = *Cryphonectria parasitica*. *R. irregularis* proteins are identified by JGI numeric codes. Protein ID (NCBI or JGI): MoAGO1 = XP_003716704.1, MoAGO2 = XP_003717504.1, MoAGO3 = XP_003714217.1, NcQDE-2 = XP_011394903.1, NcSMS-2 = EAA29350.1, SpAGO1 = O74957.1, McAGO-1 = 104,161, McAGO-2 = 195,366, McAGO-3 = 104,163, CpAGL1 = ACY36939.1, CpAGL2 = ACY36940.1, CpAGL3 = ACY36941.1, CpAGL4 = ACY36942.1. The numbers at the nodes are bootstrap values (%) for 1000 replications. ERM = extra radical mycelium, IRM = intra radical mycelium. Tree was rooted using *Arabidopsis thaliana* Argonaute 6 (NCBI Reference Sequence: NP_180853.2). Figure was generated with Evolview v2

Regarding AGO, 25 of the 40 AGO-like sequences, possessed all the 4 typical AGO core domains - piwi, PAZ, MID and N-terminal [45] - whereas the remaining 15 lacked some of the non-piwi domains present in typical AGO (Fig. 2). A phylogenetic analysis of the identified AGO-like sequences revealed that the *R. irregularis* genome encodes for 5 proteins (1580797, 1704186, 1662120, 1662010, 1697341) related to AGO of fungi belonging to Ascomycota (*M. oryzae*, *N. crassa*, *C. parasitica* and *S. pombe*), while 25 proteins (61334, 1606291, 1456683, 1478504, 1478501, 1582012, 1450356, 1741331, 1067111, 1577331, 1745457, 1764424, 1462304, 1556957, 1516785, 1600861, 1851280, 1829955, 1779081, 1748319, 1755567, 1868966, 1623940, 1782262 and 1884824) form a group with the three AGO proteins from *M. circinelloides*, a fungus which belongs to the Mucoromycota phylum that also includes AMF [1], and for which the RNAi machinery has been well characterized [13, 44].

The *AGO* gene family is divided in three paralogous groups: a widespread *AGO*-like group found in plants, animals and fungi, a Piwi-like group closely related to *Drosophila melanogaster* PIWI (P-element Induced Wimpy Testis) only found in animals, and a species-specific group (group 3 *AGO*) only found in *Caenorhabditis elegans* [46]. Interestingly, the genome of *C. elegans* also displays the highest level of *AGO* gene expansion so far reported (26 total genes; [46]). Considering that a similar degree of expansion is also observed in *R. irregularis*, we wondered if some of the identified *R. irregularis* AGO-like proteins were related with those of the animal Piwi-like group or with the ones specific of the *C. elegans* group 3. For this purpose, a phylogenetic tree derived including *D. melanogaster*, *C. elegans* and *Arabidopsis thaliana* AGO did not reveal any homologous of group 3 AGO (those specific of *C. elegans*) or of Piwi-like AGO in *R. irregularis* (Additional file 1: Figure S1).

**Fig. 3:**
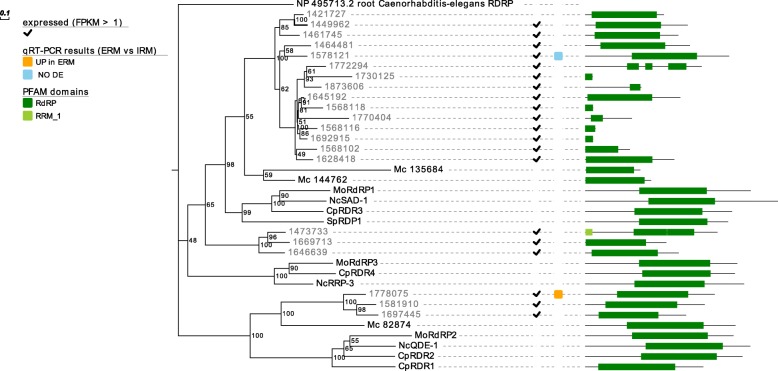
Summary of characterization of *Rhizophagus irregularis* RdRp including results of in silico gene expression, qRT-PCR, protein domains and phylogenetic analyses. Proteins are discernible by species according to a two-letter prefix: Mo = *Magnaporthe oryzae*,Nc = *Neurospora crassa*, Mc = *Mucor circinelloides*, Sp = *Schizosaccharomyces pombe*, Cp = *Cryphonectria parasitica*. *R. irregularis* proteins are identified by JGI numeric codes. Protein ID (NCBI or JGI): MoRdRP1 = XP_003721007.1, MoRdRP2 = XP_003711624.1, MoRdRP3 = XP_003712093.1, NcQDE-1 = EAA29811.1, NcSAD-1 = XP_964248.3, NcRRP-3 = XP_963405.1, SpRDP1 = NP_001342838.1, McRdRP-1 = 111,871, McRdRP-2 = 104,159, CpRDR1 = 270,014, CpRDR2 = 35,624, CpRDR3 = 10,929, CpRDR4 = 339,656. The numbers at the nodes are bootstrap values (%) for 1000 replications. ERM = extra radical mycelium, IRM = intra radical mycelium. Tree was rooted using *Caenorhabditis elegans* RdRP (NCBI Reference Sequence: NP_495713.2). Figure was generated with Evolview v2

In addition, our bioinformatics search allowed the identification of 21 putative RdRp proteins. The phylogenetic analysis shows that well characterized RdRp from Ascomycetes are grouped in three clades (Fig. 3). Fifteen *R. irregularis* proteins cluster within the clade containing RdRp1 from *Magnaporthe oryzae* and are more related to the two proteins from *M. circinelloides* (144762, 135684). This suggests that these 15 sequences may be a product of a recent gene expansion event. Three *R. irregularis* sequences (1778075, 1581910, 1697445) are grouped together with the clade containing RdRp2 from *M. oryzae*, close to the *M. circinelloides* 82,874 sequence. The association of the last three RdRp proteins (1473733, 1669713, 1646639) to the clade containing *M. oryzae* RdRp3 is not statistically well supported (Fig. 3). When we added plant RdRp from the model organism *A. thaliana* in the analysis, no difference in the structure of the tree topology was detected (Additional file 1: Figure S2).

We wondered if a similar occurrence of RNAi-related genes is noticeable in other AMF. In the recently published *Rhizophagus clarus* proteome [47] we found 2 putative DCL, 33 putative AGO-like and 17 putative RdRp. Phylogenetic analyses of *R. clarus* AGO-like, RdRp and DCL proteins revealed that they are strictly related with those of *R. irregularis* (Additional file 1: Figure S3).

**Fig. 4:**
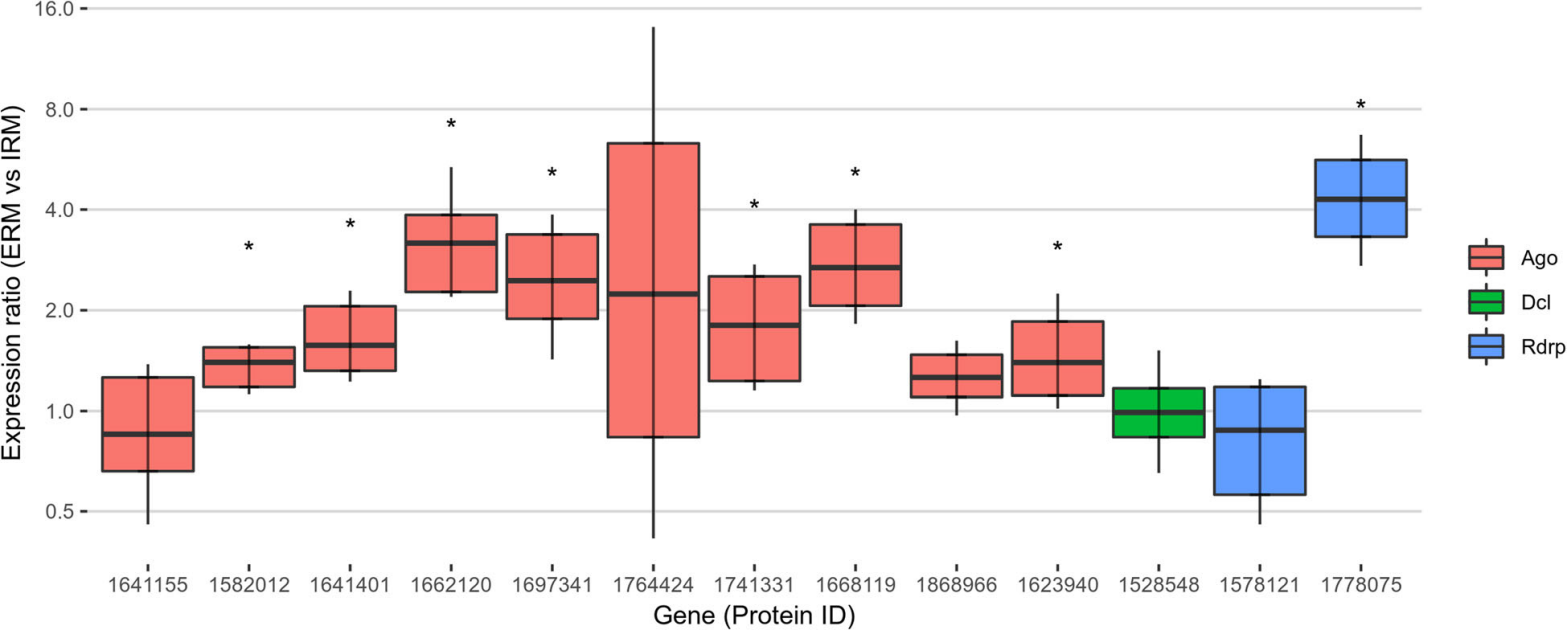
Whisker-box plot of the relative expression (ERM = “extraradical mycelium” vs IRM = “intraradical mycelium”) calculated by REST2009 software of 10 *AGO*-like, 2 *RdRp* and the *DCL* genes identified in *Rhizophagus irregularis,* here reported with their JGI protein ID. Asterisks highlight genes with significant differential expression between the two conditions

To find evidence of gene expression of the putative RNAi machinery, publicly available RNA-seq data [40] obtained from *R. irregularis* germinating spores and symbiotic tissues (mycorrhizal roots) were analyzed. Interestingly, the *DCL* gene (Fig. 1), 27 out of the 40 *AGO*-like genes (Fig. 2) and 19 out of 21 *RdRp* genes (Fig. 3) are all expressed in at least one of two considered conditions (Additional file 2). To support the in silico expression analyses, we performed quantitative RT-PCR (qRT-PCR) assays on 13 genes (the single *DCL*, 10 *AGO*-like and 2 *RdRp*), randomly chosen from the group of expressed sequences. We focused on the symbiotic phase of the *M. truncatula-R. irregularis* association, considering the extraradical mycelium (ERM) and the intraradical mycelium (IRM), obtained by removing under a stereomicroscope the ERM from mycorrhizal roots. Seven *AGO*-like and 1 *RdRp* mRNAs (1662120, 1697341, 1582012, 1741331, 1623940, 1641401, 1668119 and 1778075) were up-regulated in ERM compared to IRM, while 3 *AGO*-like, 1 *RdRp* and the *DCL* (1868966, 1764424, 1641155, 1578121 and 1528548) showed no differential expression in the two conditions tested (Fig. 4).

### Characterization of small RNAs

To characterize the *R. irregularis* sRNA population, we sequenced, with an Illumina platform, 9 sRNAs libraries prepared from biological samples in different conditions of the *R. irregularis* - *M. truncatula* symbiotic association: 3 from extraradical mycelium (ERM; fungal structures developing outside the roots after colonization), 3 from mycorrhizal roots from which we removed the extraradical mycelium (RM) and 3 from non mycorrhizal roots (RC). The presence of a functional AM symbiosis in RM samples was confirmed by qRT-PCR assays using primers for the plant AMF-inducible phosphate transporter gene (*MtPT4*) (Additional file 1: Figure S4).

**Fig. 5:**
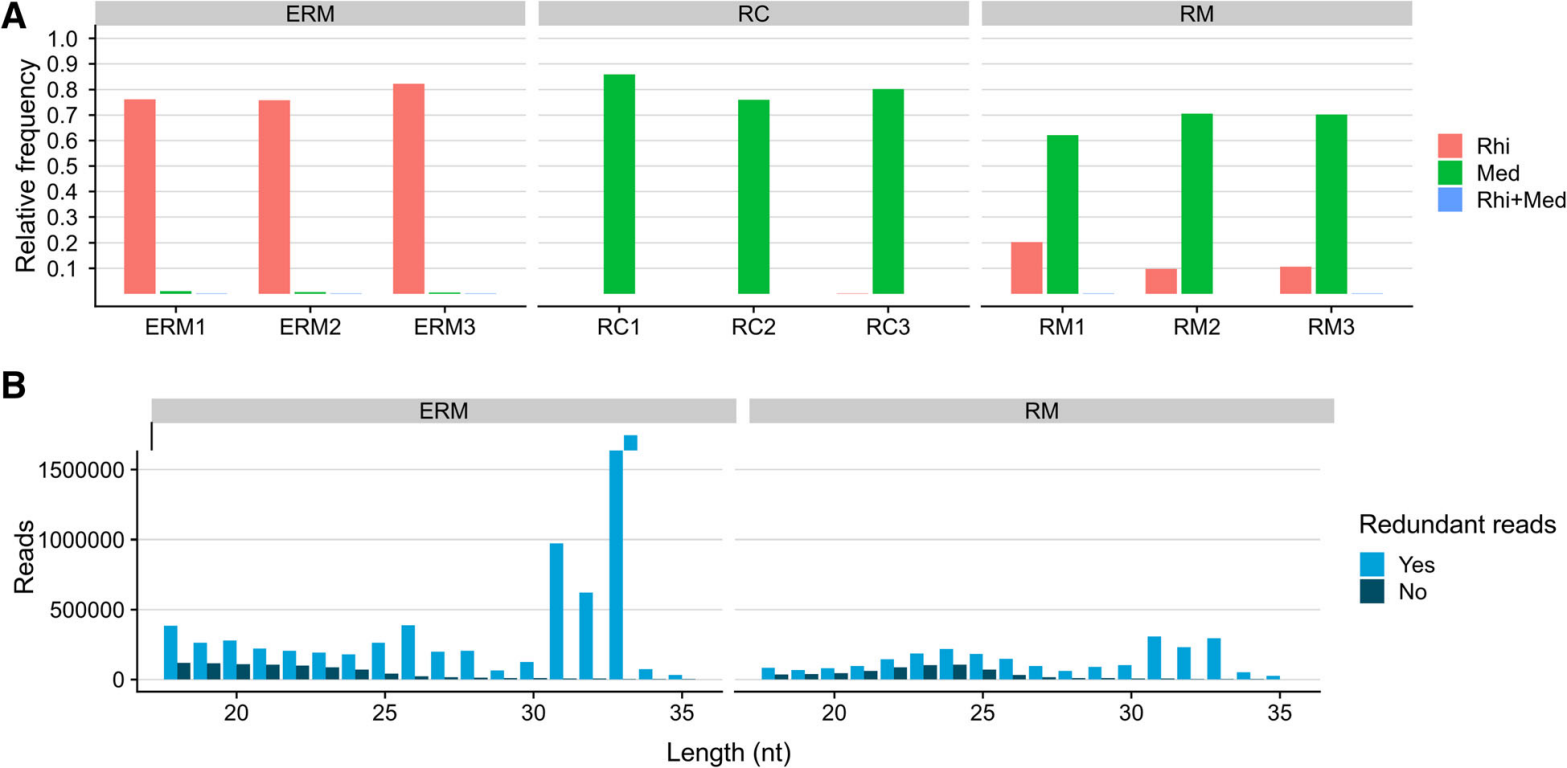
**a**) Relative mapping frequencies for each library (Rhi = reads mapping on *Rhizophagus irregularis* genome, Med = reads mapping on *Medicago truncatula* genome, Rhi + Med = reads mapping on both genomes). **b**) Length distribution (expressed in number of nucleotides) of sRNA reads (redundant and non-redundant) from ERM (extraradical mycelium) and RM (mycorrhizal roots) libraries mapping on *R. irregularis* genome

A total of 229,660,397 reads were generated; after adapter removal and filtering for quality, artifacts, tRNA, rRNA, snRNA and snoRNA presence, 53,746,056 were retained (Additional file 3). Reads were then mapped on *M. truncatula* and *R. irregularis* genomes allowing zero mismatches. Considering the different biological replicates, the 76–82% of reads from ERM libraries mapped on the fungal genome and less than 1% on the plant genome, probably because of a contamination by root material during ERM harvesting (Fig. 5a), even though we can not exclude a possible plant-originated sRNA component present natively in ERM as recently observed in the *Botrytis cinerea*-host plant interaction [27]. The 76–85% of reads from RC libraries mapped on plant genome with a very limited number of reads mapping on fungal genome (0.01–0.02%). For RM samples an intermediate situation was observed with 62–70% reads mapping on plant genome and 10–20% on fungal genome. A very low percentage of reads for each condition mapped on both plant and fungal genomes: about 0.1% for ERM, 0.01% for RC and 0.02–0.05% for RM libraries (Fig. 5a).

The evaluation of read length distribution is a useful tool to assess whether sRNAs are originated through a specific molecular pathway [48], i.e. in a Dicer-dependent manner. Plant reads present in RM and RC libraries displayed a typical enrichment of 21 and 24 nt-long sequences [37, 49], with the 21 nt-long class consisting of more redundant sequences than the 24 nt-long class (Additional file 1: Figure S5). On the contrary, the length distribution of sRNA reads from ERM and RM libraries mapping on the fungal genome (*R. irregularis* sRNAs = *Rir*-sRNAs) was bimodal with a first peak at 24 nt and 26 nt in RM and ERM respectively, and a second peak at 31–32-33 nt in both samples; the 31–33 nt long reads consist of extremely redundant sequences (Fig. 5b).

The analysis of the 5′ terminal nucleotide composition of fungal non redundant reads showed that approximately half of the sRNAs shorter than 26 nt starts with uracil (Additional file 1: Figure S6). Interestingly, in plants, 5′ U enrichment has been associated to the selective loading of sRNAs to specific AGO proteins [50]. The features of these sRNAs, together with the identification of RNAi-related genes, suggest the presence of an active sRNAs-generating pathway in *R. irregularis*.

### Characterization of *R. irregularis* sRNA-generating loci

*Rir*-sRNAs from RM and ERM libraries were used for a genome-guided sRNA-generating loci discovery and characterization, by ShortStack software [51]. Setting a cut-off of 10 RPM (reads per million reads), 2131 sRNA-generating loci, defined by the 95% of *Rir-*sRNAs, were predicted (whole characterization data in Additional file 4). Thirty three percent (702) of *Rir*-sRNA-generating loci localized in intergenic regions while the remaining 67% (1429) shared, for at least one nucleotide, the same genomic coordinates of annotated genes (protein-coding genes). We observed that 69% of *Rir*-sRNAs-generating loci overlapping with annotated genes produced sRNAs from the same strand of the overlapped genes, 8% from the opposite strand while 7%, despite being located on a specific genomic strand, were localized in regions coding for genes on both strands (so we could not assess if they are sense or anti-sense to genes). The remaining 16% of loci produced sRNAs on both genomic strands. These observations are in line with the results obtained for *M. circinelloides*, where exons were the major source of sRNAs [43]. Differential expression analysis, performed with DESeq2 [52], revealed that 225 *Rir*-sRNAs-generating loci were up-regulated in terms of sRNAs production in ERM while 589 of them were up-regulated in RM; the remaining 1317 loci were not regulated between the two conditions (Additional file 1: Figure S7A).

Considering that transposable elements are an important source for sRNA production [12], we looked for similarity of *Rir*-sRNA-generating loci with fungal repetitive elements from RepBase 23.04 [53]. A total of 236 loci, representing the 11% of the identified loci, had strong similarity with transposons: 93 with DNA transposons, 61 with LTR retrotransposons and 22 with non-LTR retrotransposons. *Rir-*sRNAs mapping on these loci were enriched in 24 nt long sequences (Additional file 1: Figure S8).

**Fig. 6:**
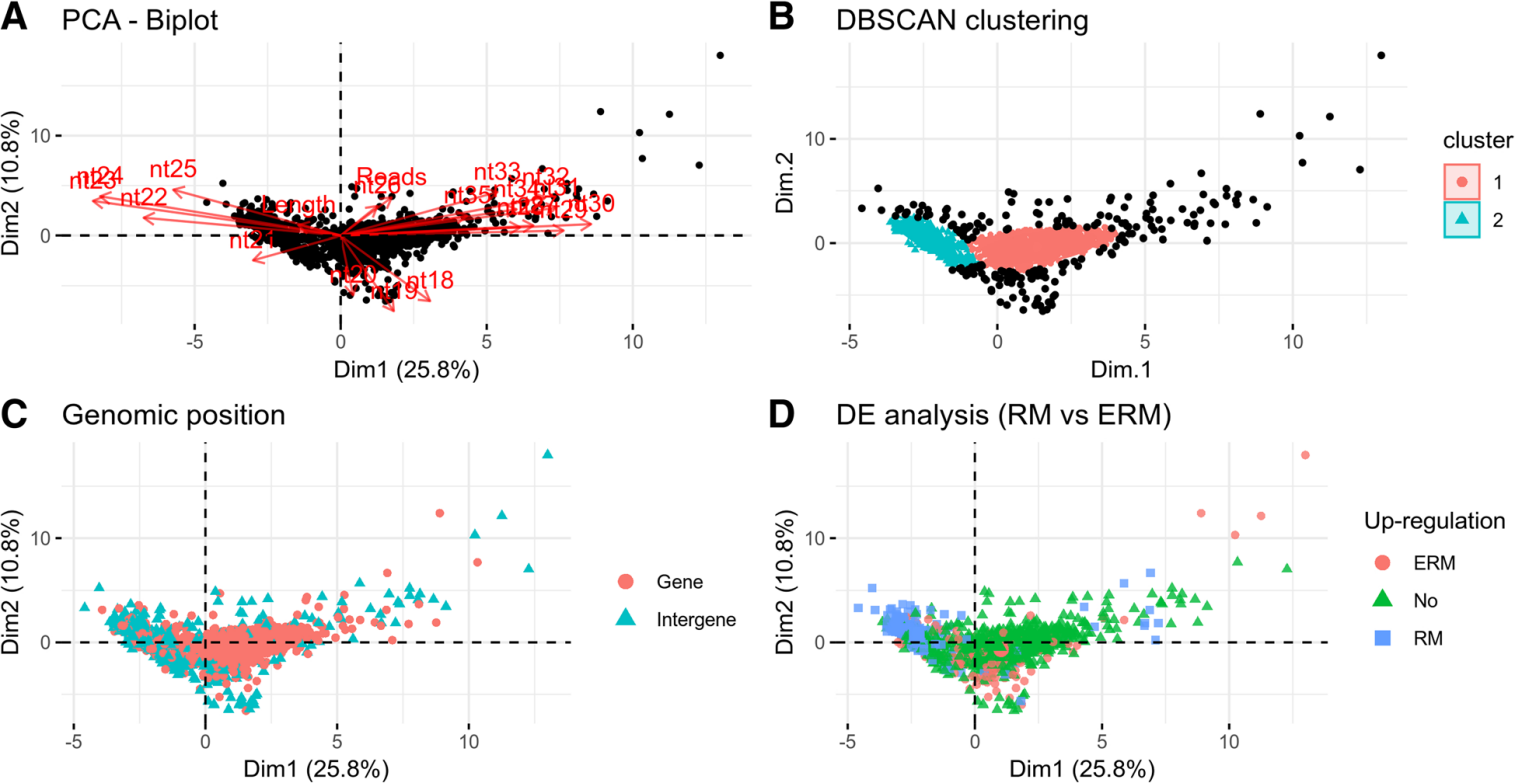
Characterization of *Rir-*sRNA generating loci. **a**) Biplot of principal component 1 and 2 of PCA based on the length of loci, the total number of mapped reads and the nucleotide size proportion of *Rir*-sRNAs (from 18 nt to 35 nt) defining each locus (19 total variables); **b**) DBSCAN clustering reveals the presence of two distinct populations of data (Cluster 1 and 2); **c**) Overview of the positions of the loci compared to those of protein-encoding genes; **d**) Differential expression analysis of loci between ERM (extra radical mycelium) and RM (mycorrhizal root) conditions (ERM = up-regulated in ERM; No = not differentially regulated; RM = up-regulated in RM)

**Fig. 7:**
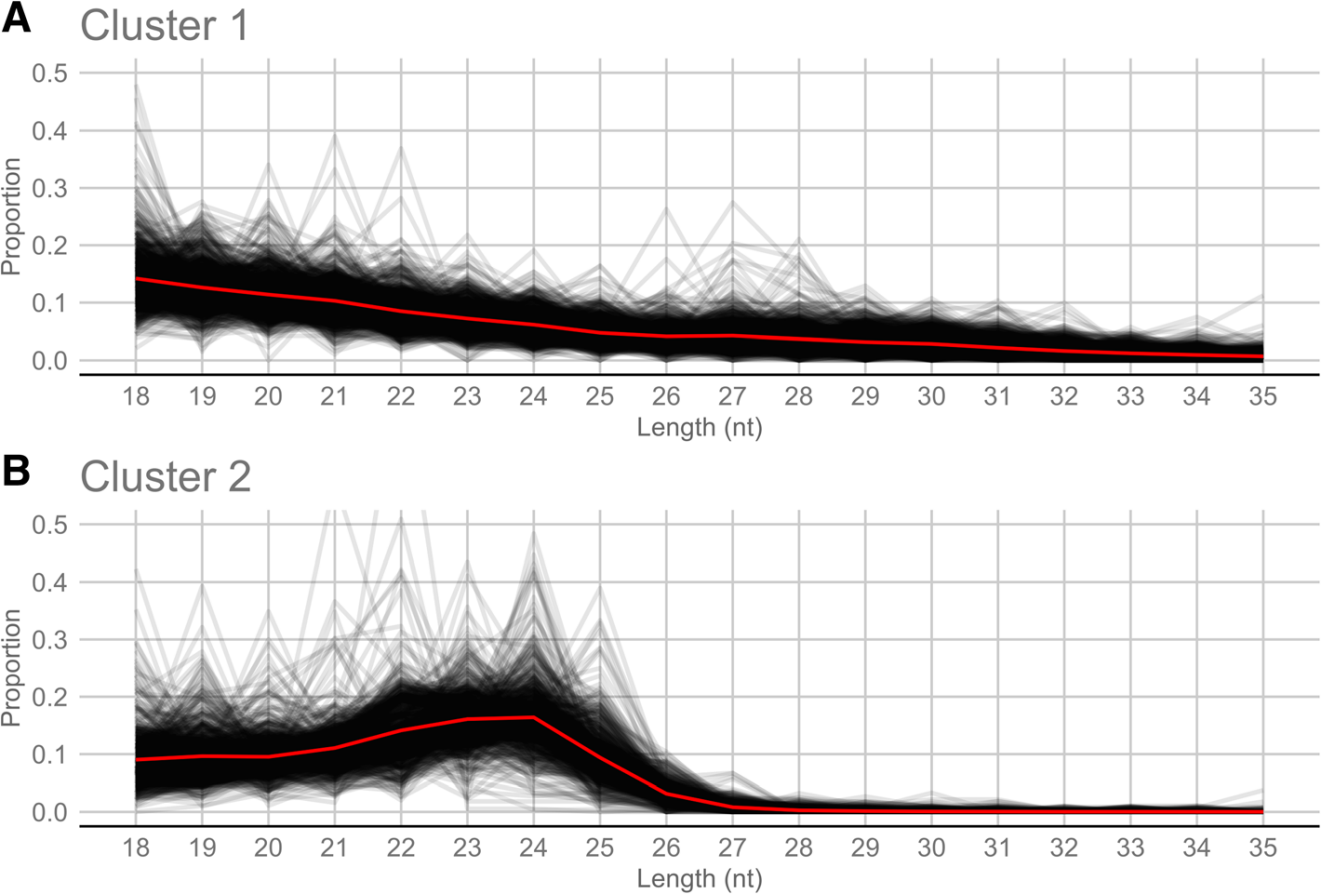
Length distribution (in number of nucleotides) of small RNA reads that defined the *Rir-*sRNA-generating loci of Cluster 1 (**a**) and 2 (**b**) according to DBSCAN clustering. Black lines are length distribution of the individual loci and red lines are the average length distribution of the loci belonging to the clusters

In order to evaluate the possible existence of different populations of *Rir*-sRNA-generating loci, we performed a Principal Component Analysis (PCA). For this purpose, as proposed by Fahlgren et al. (2013) [54], we used a model considering the following independent variables: the length of loci, the total number of mapped reads and the nucleotide size proportion of *Rir-*sRNAs (from 18 nt to 35 nt) defining each locus (19 variables in total). The first principal component (PC1), that explained the 25.8% of the total variance, mainly based on the proportion of 21–25 and 27–35 nt-long reads, differentiated the *Rir*-sRNA-generating loci in 2 groups (Fig. 6a). The use of DBSCAN (density-based spatial clustering of applications with noise) algorithm [55] confirmed indeed the presence of two different groups of data: cluster 1 and cluster 2 composed of 1100 and 819 loci, respectively (Fig. 6b). The average nucleotide size distribution of the reads for loci belonging to cluster 1 revealed a decreasing curve from 18 to 35 nt with no evident peaks while for cluster 2 we recorded an enrichment in 22–24 nt-long sequences (Fig. 7).

Interestingly, the two clusters differentiated also on the basis of the genomic positions of the *Rir*-sRNA-generating loci relative to protein-encoding annotated genes (Fig. 6c) and on the basis of the expression levels of the loci between ERM and RM conditions (Fig. 6d). In fact, 94% of *Rir*-sRNA-generating loci from cluster 1 localized in genic regions and 15 and 1% were up-regulated in ERM and RM, respectively, while 37% of loci from cluster 2 localized in genic regions and 1 and 66% were up-regulated in ERM and RM, respectively.

Finally, we observed that 4 and 16% of loci from cluster 1 and cluster 2 respectively showed homology with sequences in RepBase (21 DNA transposons, 12 LTR retrotransposons and 7 non-LTR retrotransposons for cluster 1; 69 DNA transposons, 46 LTR retrotransposons and 15 non-LTR retrotransposons for cluster 2).

### *R. irregularis* generates putative miRNA-like sequences

**Fig. 8:**
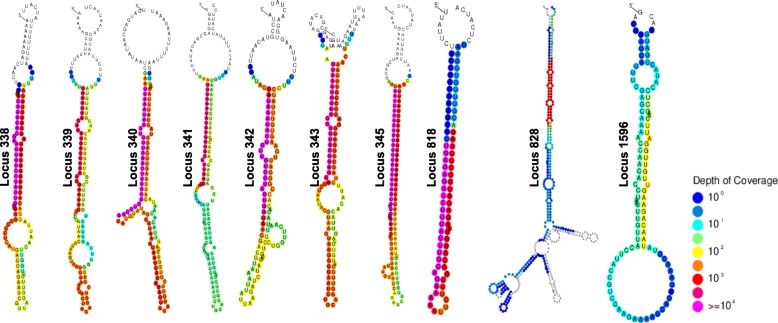
Predicted secondary structures of putative *Rhizophagus irregularis* miRNA-like with color-coded sRNA-seq coverage per nucleotide

The ShortStack software predicted 10 *Rir*-sRNA-generating loci as miRNA-like (loci 338, 339, 340, 341, 342, 343, 345, 818, 828 and 1596; Table 1). These sequences, if transcribed, have the ability to form hairpin structures and the software predicts the accumulation of miRNA-miRNA* pairs (Fig. 8). The length of these loci varies from 102 nt (locus 338) to 610 nt (locus 828) while their expression (considering the sum of all RM and ERM libraries) ranges from 196 reads (locus 1596) to 74,526 reads (locus 340; the fourth most expressed sRNA-generating locus). Interestingly, 7 loci are located on the negative strand of the same genome scaffold (scaffold 28) in sequential order within a 8.3 kbp region. The length distribution of reads produced by the *Rir-*miRNA-like loci, as well as the length of mature miRNA sequences, are enriched in sequences from 19 nt to 24 nt (data not shown). Three *Rir-*miRNA-like loci (341, 342, 828) show an increased sRNA production in RM condition.

**Table 1:** Characteristics of predicted miRNA-like loci

Genomic position	Name	Length	Reads	Strand	Mature miRNA-like	Mature miRNA-like length (nt)	Up-regulation
scaffold_28:345427–345,528	Locus_338	102	37,395	–	UAAACACGAACUGUCCUAGU	20	No
scaffold_28:346121–346,253	Locus_339	133	2778	–	UAAAUACCGCGUGACCUAGA	20	No
scaffold_28:347535–347,650	Locus_340	116	74,526	–	UUAAAUAGAUGUUGAACUUGGUG	23	No
scaffold_28:349765–349,990	Locus_341	226	3636	–	UUUAAAGAGUAGGUGUCCUGAUC	23	RM
scaffold_28:350996–351,184	Locus_342	189	9875	–	UAAACACUGCUGUCCUAGUGG	21	RM
scaffold_28:351831–351,938	Locus_343	108	7174	–	UUAAAUGGGGGGUGUACUG	19	No
scaffold_28:353658–353,769	Locus_345	112	62,117	–	AAUUAAAGUGUGGCUGUCUUGGUG	24	No
scaffold_81:287993–288,405	Locus_818	413	17,160	+	UGAGAGAUCUUUACUUGCAG	20	No
scaffold_81:370577–371,186	Locus_828	610	2143	–	CGAGGAUCGAGAGCUUGCACGUCA	24	RM
scaffold_245:162243–162,808	Locus_1596	566	196	–	UAACAGAAGUUGUUGGAUU	19	No

### Identification of *Rir-*sRNAs potentially targeting *M. truncatula* transcripts

Considering that cross-kingdom RNA silencing seems to be a quite common and widespread phenomenon [17, 21, 25], we searched for in silico evidences of *M. truncatula* mRNAs (*Mtr-*mRNAs) potentially targeted by *Rir*-sRNAs. We combined our sRNA-seq results with degradome (PARE-seq) data collected in similar experimental conditions for the *M. truncatula-R. irregularis* symbiotic association (mycorrhizal and non-mycorrhizal roots; [37]). For this purpose, we pooled together all the *Rir-*sRNAs from ERM and RM libraries and counted, for each individual sequence, the number of total occurrences. The 11,396 most abundant *Rir-*sRNAs (expression ranges from 1,945,411 reads for the most abundant *Rir*-sRNA to 19 reads for the less abundant) were used for target prediction against *M. truncatula* transcriptome followed by PARE validation using sPARTA [56].

Resulting targets were further filtered maintaining only the predictions that met the three following criteria: *Rir-*sRNA size between 21 and 24 nt (considering that plant sRNAs involved in RNAi are 21–24 nt long; [57]), adjusted *p*-value less than 0.05, at least 5 PARE reads at cleavage site in mycorrhizal condition and no reads at the same site in non-mycorrhizal one (to limit the search to cleavage signals specific for mycorrhizal condition).

This analysis identified 310 *Rir-*sRNA - *Mtr-*mRNA interactions involving 237 *Mtr-*mRNA and 274 *Rir-*sRNA (Additional file 5). These *Rir-*sRNAs were mainly produced by *Rir-*sRNA-generating loci up-regulated in RM condition (120), while the remaining corresponded to loci up-regulated in ERM condition (19) or not regulated (47) (Additional file 1: Figure S7B); 4 *Rir-*sRNAs originated from genome regions not annotated as *Rir-*sRNA-generating loci (Additional file 5). Putative targeted *Mtr-*mRNAs were enriched in 8 gene ontology (GO) terms: serine hydrolase activity (GO:0017171) and serine-type peptidase activity (GO:0008236) for the “molecular function” ontology, while cytoplasm (GO:0005737), Golgi apparatus (GO:0005794), Golgi apparatus part (GO:0044431), Golgi membrane (GO:0000139), cytoplasmic part (GO:0044444) and endomembrane system (GO:0012505) for the “cellular components” ontology.

To understand if the *Mtr-*mRNAs potentially targeted by *Rir-*sRNAs can also be targeted by endogenous plant sRNAs, we performed a target prediction and PARE validation against *M. truncatula* transcriptome using as queries the *M. truncatula* miRNAs (*Mtr*-miRNAs) (miRBase, Release 22, [58]). Considering only the predictions with adjusted p-value less than 0.05 and at least 5 PARE reads at cleavage sites in mycorrhizal condition, we identified 296 *Mtr-*miRNA / *Mtr-*mRNA interactions involving 172 *Mtr-*miRNAs and 165 *Mtr-*mRNAs (Additional file 6). According to our criteria, eleven *Mtr-*mRNAs were potentially targeted by both *Rir-*sRNAs and *Mtr-*miRNAs (AES68798, AES68809, AES88206, AES68814, AES74320, AES92729, AES98787, AET00614, KEH18078, KEH21177 and KEH27629) (Additional file 7).

Plant miRNAs (generally the 22 nt-long ones) can trigger secondary phased small interfering RNAs (siRNA) production from their target transcripts [59]. Such a phenomenon was also observed for some *Cuscuta campestris* miRNAs involved in host-gene regulation by cross-kingdom RNA silencing [28]. To understand whether *Rir*-sRNAs can trigger secondary siRNA production from their in silico predicted *Mtr-*mRNA targets, we followed the approach proposed by Sahid et al. (2018) [28]. After mapping sRNA reads from RM and RC libraries on *M. truncatula* transcriptome, we performed a differential expression analysis that resulted in 575 *Mtr-*mRNAs with an increased number of mapped sRNAs from RM libraries compared to RC ones (Additional file 8). Seven out of these 575 transcripts belong to the group of 237 *Mtr-*mRNAs previously identified as putative targets for *Rir-*sRNAs (AES67976, AES71586, AES75437, AES94149, AET03346, KEH31350 and KEH43815). According to PhaseTank, none of them produced phased siRNAs [60].

## Discussion

### *R. irregularis* is equipped with a putative RNAi machinery characterized by the expansion of AGO-like and RdRp

AMF are nowadays recognized as a crucial component of the beneficial plant microbiota. Although their nature of obligate biotrophs has been an important obstacle for their molecular characterization, recent advances in ‘omics techniques have allowed to obtain important insights on their biology and evolution [61]. The availability of genome sequences and transcriptomic data covering different fungal life stages gave us the opportunity to study a still unexplored aspect of AMF, that is the prediction of the existence of the RNAi machinery, a key platform for endogenous gene regulation and a possible source of cross-kingdom RNA silencing [10, 40, 47, 62, 63].

By means of blastp- and keywords-based searches, we identified 1 DCL, 40 AGO-like and 21 putative RdRp protein homologues in the genomic resources of the AMF *R. irregularis* [40]. Fungi typically possess only 1–2 DCL, 1–4 AGO and 1–4 RdRp [64], therefore, this high number of AGO-like and RdRp coding genes is unusual. The comparison with the phylogenetically closely related *R. clarus* helped in pointing out the level of conservation among the different AGO, RdRp and DCL proteins.

Interestingly, beside the canonical DCL protein Lee et al. (2018) identified in *R. irregularis* two additional prokaryotic (class I) ribonuclease III proteins (RIRNC2 and RIRNC3) that may arise by putative horizontal gene transfer events from cyanobacteria [30]. It would be interesting to understand whether these two proteins are also functional in the processing of dsRNA.

Concerning AGO, we identified 7 AGO-like proteins that consist of small proteins (peptides) containing only the piwi domain, and that therefore are likely not functional as the AGO protein generally involved in sRNA processing in fungi. Nevertheless, at least the genes encoding for 4 of them (1479276, 1848686, 1489847 and 1580797) are expressed at sufficiently high levels (Additional file 2) to hypothesize that they are indeed functional, possibly belonging to a new class of non-AGO but piwi domain-containing small peptides, with no evident conserved correspondence into the predicted *R. clarus* proteome. Since functional fungal AGO involved in RNAi have at least 5 or 6 domains (see Fig. 2), we can conclude that *R. irregularis* may possess 25 complete AGO. Among them, 5 (61334, 1606291, 1456683, 1478504, 1478501) do not have specific homologs in the *R. clarus* genome, thus representing a species-specific subclade; however, at the moment there is no evidence of corresponding expressed sequences in transcriptomic databases (Additional file 2).

Comparing both *Rhizophagus* species and *M. circinelloides*, our AGO phylogenetic analysis reveals another interesting aspect: both *Rhizophagus* species have a group of 5–6 AGO-like proteins that are in a clade with well-characterized AGO from ascomycetes, whereas *M. circinelloides* seem to have lost such AGO. The three *M. circinelloides* AGO are in a well-supported clade with 25 AGO-like proteins from *Rhizophagus* displaying a specific AMF expansion of such AGO-like clade. Wider comparisons that include AGO from other eukaryotic kingdoms (plants and animals in specific) help to exclude that any of the fungal AGO are homologues of the PIWI-AGO present in insects (piwi and aubergine from *Drosophila melanogaster*) and point to a specific expansion of AMF specific AGO-like protein clade, well separated from plant and animal AGO.

Regarding RdRp, the discrepancy in the number of RdRp between our work and Lee et al. (2018) -21 vs 3- is likely due to the different criteria used to identify RdRp: while we looked for proteins showing the presence of an RdRp domain, Lee et al. (2018) used rather restrictive parameters to find homologs of RdRp proteins characterized from other fungi (but excluding other RdRp more specific of AMF fungi) [30]. Indeed, following our approach, a similar number of RdRp (17) were also found in the *R. clarus* predicted proteome confirming the conservation of this group of AMF-specific and RdRp motif- containing proteins.

In analogy to what happens with AGO-like, at least 6, out of the 21 identified, are constituted of small peptides that have only the conserved RdRp domains (1730125, 1645192, 1568118, 1770404, 1568116, and 1692915): in this case, all 6 are consistently expressed in transcriptome datasets, and one of them has a specific homologue in *R. clarus*. Also in this case, we can hypothesize the existence of a number of functional RdRp domain-carrying small proteins, so far undescribed in other fungi.

In the case of the phylogenetic tree that includes plant RdRp, it is apparent that orthologues of AMF, fungal and plants are present, and only a subset of RdRp are specific for AMF (Additional file 1: Figure S2).

Overall, the expansion of AMF-specific AGO-like and RdRp protein gene families prevents us from drawing any conclusion based on functional homology to animal, fungal or plant proteins containing the same conserved domains. In this respect, we can hypothesize that some of these proteins are not involved in RNAi or RNA silencing pathways, but might have a completely new set of functions even unrelated to RNA processing. Expansion of gene families is often accompanied by functional differentiation and gene expression fine-tuning. In this respect, it is tempting to correlate AGO-like and RdRp expansion in AMF to their large genomes, rich in repetitive DNA, mainly transposable elements. This expansion could be the result of a co-evolution between specific anti-transposable element defense and a diversity of transposons present in the genome. It is worth to mention that an evolutionary related AGO enzyme in prokaryotes was shown to protect its host against mobile genetic elements through DNA-guided DNA interference [65]. The setup of a reverse genetic system (i.e. HIGS and VIGS) based on delivery of dsRNA or sRNA for AMF genes would possibly help understanding some of the functional specificities associated to each of the AGO-like or RdRp present in the genome.

In silico transcriptome analyses provided evidence of gene expression for the majority (*DCL*, 27 out of the 40 *AGO*-like and 19 out of 21 *RdRp* sequences) of the identified RNAi-related genes. Moreover, targeted gene expression profiles showed that some *AGO*-like and *RdRp* genes are differentially expressed between the extraradical and the intraradical mycelium two functionally distinct compartments of the symbiotic phase at the interface with the soil and with the plant, respectively, supporting the interesting possibility of a functional differentiation among distinct *AGO*-like and *RdRp* genes in the control of the symbiotic process.

### *R. irregularis* sRNAome is characterized by 2 different populations of *Rir*-sRNA-generating loci and by the existence of miRNA-like sequences

Small RNAseq data generated for the three biological conditions (ERM, RM and RC) were of good quality with relatively high genome-mapping percentages. As expected, enrichment of 21 and 24 nt-long sequences was observed in plant reads of RM and RC samples [37, 49] which proofs the good quality of the sRNA and corresponding libraries preparations. Here the attention focused to the fungal sRNAome as no data are currently available for AMF. The length distribution of *R. irregularis* sRNAs (*Rir*-sRNAs) with two distinct peaks clearly differs from a flat curve over 20 nt typically observed in organisms not provided with RNAi machinery [16] or fungal *dcl* knock-out mutants [66].

The characterization of *Rir*-sRNA-generating loci suggests that *R. irregularis* possesses at least two different populations of sRNA-generating loci: the first one (cluster 1) mostly includes sequences overlapping with protein-coding genes, mainly not differentially expressed between ERM and RM, and that produce sRNAs of different sizes, while the second one (cluster 2) is enriched in 22–24 nt-long sRNAs from intergenic sequences, mainly up-regulated in RM. Based on these results, we hypothesize that at least two distinct molecular pathways could contribute to the production of *Rir-*sRNAs. Indeed, two pathways are involved in sRNA generation in *M. circinelloides*: a Dicer-dependent and a Dicer-independent but RdRp-dependent one. Moreover, in analogy to what we observed, protein-encoding genes are the major source for sRNAs production in *M. circinelloides* [13, 44, 67]. Since in both *R. irregularis* and *M. circinelloides* the protein-encoding genes are the major source for sRNA, we could speculate that the *Rir-*sRNA originated from protein-coding sequences are involved in post-transcriptional regulation of the gene from which they originate, as it has been demonstrated in *M. circinelloides* [68, 69]*.* We did not perform a Rir-sRNA target prediction on fungal endogenous genes since no software for sRNAs target prediction in fungi is available and the predictions made with software developed for other organisms have rarely been experimentally validated [13].

Among the population of *Rir*-sRNA-generating loci, 10 were predicted as miRNA-like. So far, miRNA-like have been identified in several fungi belonging to Ascomycota and Basidiomycota phyla but not in the basal fungus *M. circinelloides* [13, 69, 70]. This is the first evidence of miRNA-like occurrence in the Mucoromycota group. Since no miRNA-like database is currently available for fungal sequences, analysis for homolog sequences could not be done in an automated way. Remarkably, based on the current literature, unlike other eukaryotes, miRNA-like sequences are not conserved among fungi belonging to different genera [70]. Interestingly, three miRNA-like are up-regulated in the intraradical phase, which could lead to the hypothesis of miRNA-like AMF genes required to manipulate fungal or host plant gene expression; however, further analyses are necessary to confirm their possible functional role.

### In silico evidences of *M. truncatula* mRNAs potentially targeted by *Rir*-sRNAs

In the absence of a tool specifically designed for the cross-kingdom RNA silencing towards plant transcripts, we have used sPARTA as one of the most powerful tool for target prediction and PARE validation in plants. Our in silico analysis, supported by degradome data, predicted 237 plant genes as putative targets of specific fungal sRNAs. Functional categories associated to these genes shows an enrichment in the GO terms related to hydrolases and endomembrane systems. Some specific target genes are here discussed in detail for their possible role in the AM symbiosis. The predicted target gene encoding the Specific Tissue (ST) protein 6 (transcript ID: AES73699) belongs to the family of ST proteins, whose function is unknown, but for which transcriptomic data suggest an involvement in biotic and abiotic stress [71, 72]. This protein family has been described in Fabaceae and Asteraceae but seems to be absent in others plant groups such as Brassicaceae [72]. Transcriptomic data suggest their involvement in biotic and abiotic stress [71, 72]. Interestingly, the expression of *M. truncatula ST6* (*Mt*ST6) gene was found to be modulated during the different steps of the AM association [73, 74] and is induced by fungal diffusible signals, during hyphopodium formation [75] and in arbusculated cells [74]. In this regard, we speculate that one identified *Rir-*sRNA (3121–59) might have a regulatory role on *MtST6* gene expression during the intraradical phase.

Another predicted target is the Responsive To Dehydration 22 (RD22; transcript ID: AES74153) gene, an ABA-dependent signaling gene involved in abiotic stress tolerance [76–78] and in pathogen susceptibility ([79] and references therein). ABA positively regulates AM symbiosis; however, contradictory results have been obtained on ABA content in mycorrhizal roots [80]. In tomato, a gene involved in ABA catabolism (CYP707A3) was specifically expressed in arbuscule-containing cells, while the gene *SlNCED*, involved in the ABA biosynthesis, was detected only in cortical cells from non mycorrhizal plants, suggesting that a balance between biosynthesis and catabolism of ABA is determinant for the differentiation of arbuscules [81]. In this context, the targeting of the RD22 gene by three *Rir-*sRNAs (773–218, 10,035–21, 10,035–21) could be related to the down-regulation of this gene in arbusculated cells [74].

Interestingly, among the putative target genes of *Rir*-siRNAs, identified in this study, one encodes a DREPP plasma membrane protein (MtDREPP) (transcript ID: KEH37321) which was found to be down-regulated in mycorrhizal roots compared to non-mycorrhizal roots [82]. Host roots undergo plasma membrane (PM) remodeling events during the AMF colonization process from initial contact to intracellular accommodation of fungal structures [83]. In particular, arbuscule accommodation requires both PM expansion and periarbuscular membrane (PAM) generation. These events, that lead to dynamic change of PM protein composition [82] and polarized secretion mediated by exocytotic fusion of membrane vesicles [2], might involve MtDREPP modulation.

It is worth mentioning that, among the *Mt*-mRNAs putative targets, we identified the AM-induced *MtVapyrin* (transcript ID: KEH25576) gene (Ankyrin repeat RF-like protein), which is required for arbuscule development [84, 85] and PAM formation [86]. It is tempting to speculate that its predicted targeting *Rir-*sRNA (2559–70) could contribute to modulate *MtVapyrin* expression in different cell populations and/or during arbuscule formation.

Non-specific phospholipase C4 (*NPC4*) (transcript ID: KEH18078) is another predicted *Rir-*sRNA target gene whose gene product is localized to the PM. It shows high homology with Arabidopsis NPC4, a phospholipid-cleaving enzyme responsible for lipid remodeling during phosphate-limiting conditions. In *Arabidopsis*, it has been demonstrated that this gene family could be involved in plant defense response against different pathogens playing a role not only in elicitor recognition processes, but also in downstream disease resistance signaling [87].

Another interesting putative target gene involved in host defense response is a nuclear-binding leucine-rich repeat (NBS-LRR) type disease resistance encoding gene (transcript ID: AES68798) which shows high similarity with rice *OsRGA3*, a *Resistance* (*R*) gene associated with rice blast resistance. Since the response of plants to AMF involves a transient and spatial activation of defense mechanisms [88] the *Rir-*sRNA (7710–27) could be responsible for repressing this gene to allow AMF colonization.

Although supported by computational analyses, further work is needed to experimentally confirm these putative *Rir-*sRNA-*Mtr-*mRNA interactions: 5′ RACE assays would be useful to further validate cleavage sites and co- expression of sRNA and its putative mRNA target in transient transformation assay would be helpful to verify the existence of a selective RNAi in vivo [89].

## Conclusions

The description of RNAi-related genes, showing an expansion of *AGO*-like and *RdRp* genes, and the characterization of the sRNA population indicate that *R. irregularis* is equipped with a functional sRNA-generating pathway. Our in silico analysis predicted 237 plant genes as putative targets of specific fungal sRNAs suggesting that a cross-kingdom post-transcriptional gene silencing may occur during AMF colonization.

Since HIGS and VIGS tools have been shown to function on AMF, it is likely that interspecies RNA movement also occurs from host plant towards AMF: the dataset generated in this work can be exploited to further investigate plant to fungus RNA exchanges in the AM symbiosis.

## Methods

### Biological material and growth conditions

All the fungal material (*R. irregularis* DAOM 197198) was obtained from mycorrhizal association with *Medicago truncatula* (Jemalong A17) plants. Nine day old *M. truncatula* seedlings, germinated in sterile conditions, were inoculated, using the Millipore sandwich method [90], with extraradical fungal structures (ERM) obtained from 2 in vitro monoxenic cultures of *Agrobacterium rhizogenes*-transformed chicory roots in two-compartment Petri plates [91]. In parallel, control non-mycorrhizal plants were treated in the same way, but avoiding the addition of the fungal inoculum. All the plants were fertilized with Long Ashton nutrient solution containing 32 μM KH2PO4 and grown in a climate-controlled room at 22 °C with a photoperiod of 14-h light and 10-h dark. After 60 days from the inoculum, plant and fungal materials were harvested. The ERM was manually collected with tweezers under a stereo microscope. Mycorrhizal roots, from which the ERM was removed, were then collected and considered as the intra-radical mycelium (IRM, for qRT-PCR experiment) or mycorrhizal roots (RM, for RNA-seq analysis). Non mycorrhizal roots (RC) were observed under stereomicroscope, to confirm the absence of fungal structures, prior to collection. The harvested material was immediately frozen in liquid nitrogen, lyophilized and stored at − 80 °C.

### Identification of RdRp, DCL and AGO homologs

We screened, by keywords searches, the recent release of *R. irregularis* genome [40] for the presence of the putative homologs of fungal DCL, RdRp and AGO genes on JGI MycCosm portal [41]. For the identification of AGO sequences, we searched for “piwi” and we retrieved (and considered as AGO-like) all the genes with a Piwi domain (Pfam family: PF02171). Regarding RdRp we retrieved all the genes annotated with a KOG (EuKaryotic Orthologous Groups) ID: KOG0988 (“RNA-directed RNA polymerase QDE-1 required for post-transcriptional gene silencing and RNA interference”). For DCL we searched for “dicer” and we kept only the sequences with two RNAse III domains [42].

To identify the RNAi-related homologs genes in *R. clarus*, we first aligned separately AGO-like, RdRp and DCL *R. irregularis* proteins with MAFFT v7.310 (option: --auto) [92] and the alignments were used to build profiles HMM with hmmbuild with default parameters (HMMER 3.1b2 [93]). Then, using hmmsearch (HMMER 3.1b2) with default parameters, we searched for homologs in *R. clarus* proteome [47]*.* At that point, the resulting sequences were searched for protein domains with hmmscan (options: --cut_ga --domtblout; HMMER 3.1b2) against Pfam-A version 32.0 [94] HMM profiles and then we kept the sequences with a “Piwi” domain for AGO-like, a “RdRP” domain for RdRp and two “Ribonuclease_3” domains for DCL.

### Phylogenetic analyses

The whole amino acid sequences of DCL, AGO and RdRp genes were aligned with MAFFT v7.310 (option: --auto) [92]; the alignments were used for phylogenetic inference by the Maximum Likelihood method implemented in the IQ-TREE software (options: -m TEST -bb 1000 -alrt 1000) [95]. The software performed model selection [96], tree reconstruction and branch support analysis by ultra-fast bootstrap method [97] (1000 replicates). Trees were visualized with Evolview v2 [98]. Protein domains annotations (for tree visualization) were retrieved using hmmscan (options: --cut_ga --domtblout; HMMER 3.1b2) against Pfam-A version 32.0 [94].

### In silico gene expression analysis

We retrieved the cDNA of each predicted DCL, AGO-like and RdRp proteins to perform an in silico gene expression analyses, exploiting RNA-seq datasets [40] available at the NCBI Sequence Read Archive (SRR3285893–SRR3285895: 2 day germinating spores; SRR3285917–SRR3285919: symbiotic tissues). Paired reads were trimmed for adapters, filtered for qualities and aligned on cDNA with Bowtie2 (default parameters) [99]. For each sequence we calculated FPKM and we considered expressed all those with a value > 1 (arbitrary selected cut-off) in at least one of the two conditions (germinating spores and symbiotic tissues).

### RNA extraction and qRT-PCR assays

Total RNA was extracted with the RNeasy Plant Mini Kit (Qiagen) and then treated with TURBO™ DNase (Ambion). The RNA samples were routinely checked for DNA contamination by PCR analysis, using primers for *MtTef* (RM samples) and for *RiTef* (ERM samples). For cDNA synthesis about 500 ng of total RNA were denatured at 65 °C for 5 min and then reverse-transcribed at 25 °C for 10 min, 42 °C for 50 min and 70 °C for 15 min in a final volume of 20 μl containing 10 μM random hexamers, 0.5 mM dNTPs, 4 μl 5X buffer, 2 μl 0.1 M DTT and 1 μl Super-ScriptII (Invitrogen). qRT-PCR experiments were carried out in a final volume of 15 μl containing 7.5 μl of iTaq™ Universal SYBR. Green Supermix (Bio-Rad), 5.5 μl of 0.8 M primer mix and 2 μl of 1:10 diluted cDNA. Amplification were run in a Rotor-Gene Q apparatus (Qiagen) using the following program: 5 min pre-incubation at 95 °C and 40 cycles of 30 s at 95 °C, 30 s at 60–64 °C. Each amplification was followed by melting curve analysis (60–94 °C) with a heating rate of 0.5 °C every 15 s. All reactions were performed on at least four biological replicates each with two technical replicates. Relative expression and statistical analyses were performed by REST2009 [100], using as reference genes *Ri-Tef* and *Ri-BetaTubulin1* (for *R. irregularis* RNAi-related gene expression). The presence of a functional AM symbiosis was evaluated (for small RNA-seq experiment) comparing the expression of *the MtPT4* gene relative to the *MtTEF* housekeeping gene in RM (mycorrhizal roots) and RC (control non-mycorrhizal roots) samples. All primers were previously tested in conventional PCR assays on cDNA, followed by agarose gel electrophoresis, to confirm the specificity and amplification of a single fragment. The list of primers is given in Additional file 9.

### RNA extraction for sRNA-seq

For sRNA sequencing, total RNA was extracted with Direct-zol™ RNA MiniPrep (Zymo Research) kit. The concentration and quality of the nucleic acids were assessed with a Nanodrop1000 (Thermo Scientific). Samples were sent to Macrogen (South Korea) for RNA integrity check, library preparations and sequencing. A total of 9 libraries were sequenced: 3 for ERM samples, 3 for RM samples and 3 for RC samples. Each sample was a pool of equal RNA amounts from 3 different biological samples.

### Bioinformatics pipeline

Raw sRNA-seq reads, after being checked for quality with FastQC (Babraham Bioinformatics) [101], were cleaned for adapters (TGGAATTCTCGGGTGCCAAGG), artifacts (default parameters) and low quality reads (−q 28 -p 50) with Fastx Toolkit (Hannon Lab) [102]. We then removed all the reads mapping on tRNA, rRNA, snRNA and snoRNA on Rfam 12.0 database [103] (Rfam families IDs in Additional file 3) using bowtie aligner [104] allowing up to 1 mismatch. We further filtered reads removing those mapping with 0 mismatch on “ribosomal RNA” sequences of the genus “Rhizophagus” in GenBankand retained only the reads with a length between 18 and 35 nt. Nucleotide length distribution, 5′ terminal nucleotide composition and reads redundancy analyses were performed with a set of Perl and R scripts. Reads were mapped on *R. irregularis* DAOM 197198 v2.0 genome on JGI Genome Portal [105] and on *M. truncatula* A17 v4.0 genome on Ensembl [106] with 0 mismatch using bowtie.

Reads mapping on *R. irregularis* genome from ERM and RM libraries were analyzed together in a single run with ShortStack v.3.8.5 [51], for the genome-guided sRNA-generating loci prediction (options: --mismatches 0 --foldsize 1000 --dicermin 18 --dicermax 35 --pad 200 --mincov 10.0rpmm). The software produced a count table file (with number of reads from each library that defined each locus) that was used for DE analysis between ERM and RM with DESeq2 1.18.1 Bioconductor package [52]. We considered, as differentially expressed, the loci with adjusted *p*-value < 0.05 (Benjamini–Hochberg procedure). ShortStack was also used to produce an annotation file with genomic coordinates of sRNA-generating loci that was used for comparison with *R. irregularis* DAOM 197198 v2.0 gene annotation file with BEDTools [107]. To annotate a *Rir*-sRNA-generating locus on a specific genomic strand it should originate 80% of reads from the same strand (default parameter in ShortStack). Homology analysis of *Rir*-sRNA-generating loci with fungal repetitive elements from RepBase 23.04 [53] was performed with tblastx [108] (E-value <= 0.005).

For PCA we calculated the nucleotide size proportion of sRNAs for each sRNA-generating locus from 18 to 35 nt (compared to the total of sRNA reads that defined that locus) starting from ShortStack output file and we associated these data with nucleotide length of each locus and total number of reads that defined it. PCA was performed in R with “FactoMineR” (v1.41) package and results visualized with “factoextra” (v1.0.5) package. DBSCAN clustering was performed with fpc package (parameters: eps = 0.5, minPts = 30).

MicroRNA-like loci were annotated by ShortStack and their secondary structure were predicted and visualized with StrucVis v.0.3 [109].

To identify *M. truncatula* transcripts potentially targeted by *Rir-*sRNAs in a hypothetical cross-kingdom RNA silencing process, we used sPARTA v.1.20 [56], a software for target prediction and PARE validation previously used for plant datasets, as it is our experimental system for target sequences. We used the 11,396 most expressed *Rir-*sRNAs (merging all the reads from ERM and RM libraries that mapped with 0 mismatch on *R. irregularis* genome) to find targets in *M. truncatula* A17 v.4.0 cDNAs (options: -tarPred E -tarScore --tag2FASTA --map2DD –validate). For PARE validation we used published PARE-seq data (SRA accessions: SRR088877, SRR088878) obtained in similar experimental conditions (*M. truncatula-R. irregularis* symbiotic association; mycorrhizal and non-mycorrhizal roots; [37]), after being cleaned for adapters and artifacts (default parameters) and filtered for quality (−q20) with Fastx Toolkit [102]. The output of sPARTA was filtered to keep only the the *Rir-*sRNA-mRNA pairs for which: a) corrected *p*-values < 0.05 (calculated by the software as the confidence score of a sRNA-target interaction corrected for the noise around the cleavage site); b) sRNA length between 21 nt and 24 nt; c) at least 5 PARE reads at cleavage sites from mycorrhizal PARE library; d) no PARE reads at cleavage site from non-mycorrhizal PARE library.

To identify *M. truncatula* transcripts targeted by endogenous sRNAs, we used sPARTA as described above, using as queries the *M. truncatula* miRNAs (*Mtr-*miRNA) from miRBase (Release 22) [58]. The output was filtered to keep only the the *Mtr-*miRNA-mRNA pairs for which the following two conditions were both met: a) corrected p-values < 0.05; b) at least 5 PARE reads at cleavage sites from mycorrhizal PARE library.

GO enrichment analysis was performed on transcripts identified as potential targets of *Rir*-sRNAs with AgriGO [110] (p-value < 0.01; statistical test: Fisher’s test with Yekutieli multi-test adjustment method).

The analysis of secondary siRNAs production from *Mtr-*mRNAs potentially targeted by *Rir*-sRNAs was performed following the procedure applied by Sahid et al. (2018) [28]. Reads from RM and RC libraries were mapped on *M. truncatula* A17 v.4.0 genome using ShortStack v.3.8.5 (−mismatches 0,–nohp), defining the full length of each mRNA as a locus (option -locifile). The output count table file was used for DE analysis (RM vs RC) with DESeq2 v.1.18.1 as described above. The resulting *Mtr-*mRNAs with an increased number of mapped reads in RM compared to RC were also checked for their presence in the list of potential targets for *Rir-*sRNAs and those in common were analyzed for pahsiRNA production with PhaseTank v1.0 [60] (default parameters).

## Additional files

Additional file 1: **Figure S1.** Phylogenetic relationship of AGO proteins in different organisms. **Figure S2.** Phylogenetic relationship of RdRp proteins in different organisms. **Figure S3.** Phylogenetic relationship of AGO, RdRp and DCL proteins in different fungi. **Figure S4.** Expression of *MtPT4* relative to *MtTEF* assessed by qRT-PCR in RM samples (mycorrhizal roots) compared to RC ones (nonmycorrhizal roots). Data for each condition are presented as mean ± standard error. **Figure S5.** Length distribution (expressed in nucleotide) of sRNAs reads (redundant and non-redundant) from RC (non mycorrhizal roots) and RM (mycorrhizal roots) libraries mapping on *Medicago truncatula* genome. **Figure S6.** Relative nucleotide frequency of 5’ end of sRNAs reads (redundant and non-redundant) from RM (mycorrhizal roots) and ERM (extra radical mycelium) libraries mapping on *Rhizophagus irregularis* genome. **Figure S7.** Volcano plots (fold changes vs adjusted p-values) of *Rir-*sRNA-generating loci. **Figure S8.** Length distribution (in nucleotide) of sRNA reads that defined the *Rir-*sRNAs-generating loci homologous to repetitive elements in RepBase. Black lines are the length distribution of the individual loci and red line is the average length distribution of the plotted loci. (PDF 3262 kb)

Additional file 2: In silico expression analysis of AGO-like, RdRp and DCL identified in *R. irregularis.* For each sequence we reported: the protein and transcript IDs (JGI), the number of paired mapped reads and FPKM from RNA-seq data-sets (SRR3285893–SRR3285895: 2 day germinating spores; SRR3285917–SRR3285919: symbiotic tissues; Chen et al. 2018) taking into account, the nucleotide length of transcript and the amino-acid length of protein. (XLSX 9 kb)

Additional file 3: Number of sRNA reads after each filtering step and RFAM families IDs (version 12.0) used for rRNA, tRNA, snRNA and snoRNA removal. (XLSX 16 kb)

Additional file 4: Characterization of sRNA-generating loci. For each of the predicted 2131 sRNA-generating loci we reported: the name, the genome coordinates, the miRNA prediction result, the possible overlap with protein-coding genes (reported with JGI protein ID), the differential expression analysis results of differential expression analysis, the length, the number of mapped sRNA reads, the eventual ID of homologous loci from RepBase (when available), the results of DBSCAN clustering, the proportion of mapped sRNA reads of each length (from 18 nt to 35 nt). (XLSX 442 kb)

Additional file 5: Results of *Rir*-sRNA – *Mtr-*mRNA target prediction performed with sPARTA. We added at the filtered software output file the information about the number of sRNA reads, the length of sRNAs (nt),the description of targets and the names of the sRNA-generating loci that produce the sRNAs. (XLSX 47 kb)

Additional file 6: Results of *Mtr*-miRNA – *Mtr-*mRNA target prediction performed with sPARTA. We added the description of targets to the filtered software output file. (XLSX 35 kb)

Additional file 7: Prediction results for Mtr-mRNAs that are targeted by both Rir-sRNAs and Mtr-miRNAs according to sPARTA analysis. (XLSX 9 kb)

Additional file 8: Results of DE analysis of sRNA reads originated from *Mtr-*mRNAs for identification of mycorrhizal induced secondary siRNAs. We reported only the transcripts with a significant increase in number of mapped sRNAs reads in RM (mycorrhizal roots) compared to RC (non-mycorrhizal roots) condition (log2FoldChange > 0, padj < 0.05; DESeq2). (XLSX 55 kb)

Additional file 9: List of oligonucleotide used in qRT-PCR assays. (XLSX 46 kb)

## Abbreviations

AMF: Arbuscular Mycorrhizal Fungi
ERM: Extra radical mycelium
GO: Gene Ontology
RC: Non-mycorrhizal roots
RM: Mycorrhizal roots
sRNA: Small RNA
