# Supplementary material for: In silico analysis of fungal small RNA accumulation reveals putative plant mRNA targets in the symbiosis between an arbuscular mycorrhizal fungus and its host plant
Source: BMC Genomics. 2019 Mar 4;20:169. doi: 10.1186/s12864-019-5561-0 (PMC6399891; doi:10.1186/s12864-019-5561-0)
Supplement: Supplementary file 1 — Figure S1. Phylogenetic relationship of AGO proteins in different organisms. Figure S2. Phylogenetic relationship of RdRp proteins in different organisms. Figure S3. Phylogenetic relationship of AGO, RdRp and DCL proteins in different fungi. Figure S4. Expression of MtPT4 relative to MtTEF assessed by qRT-PCR in RM samples (mycorrhizal roots) compared to RC ones (nonmycorrhizal roots). Data for each condition are presented as mean ± standard error. Figure S5. Length distribution (expressed in nucleotide) of sRNAs reads (redundant and non-redundant) from RC (non mycorrhizal roots) and RM (mycorrhizal roots) libraries mapping on Medicago truncatula genome. Figure S6. Relative nucleotide frequency of 5’ end of sRNAs reads (redundant and non-redundant) from RM (mycorrhizal roots) and ERM (extra radical mycelium) libraries mapping on Rhizophagus irregularis genome. Figure S7. Volcano plots (fold changes vs adjusted p-values) of Rir-sRNA-generating loci. Figure S8. Length distribution (in nucleotide) of sRNA reads that defined the Rir-sRNAs-generating loci homologous to repetitive elements in RepBase. Black lines are the length distribution of the individual loci and red line is the average length distribution of the plotted loci. (PDF 3262 kb) [file 12864_2019_5561_MOESM1_ESM.pdf]

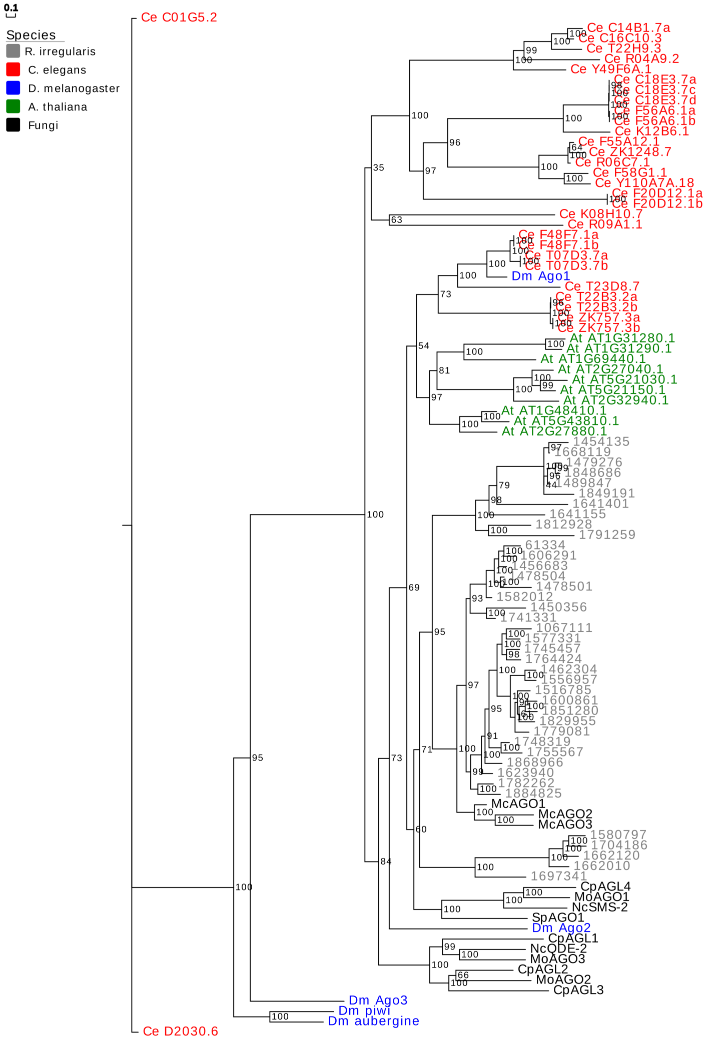

Figure S1. Phylogenetic relationship of AGO proteins in different organisms. Proteins are discernible by species according to color and a two-letter prefix: Mo = *Magnaporthe oryzae*,Nc = *Neurospora crassa*,Mc = *Mucor circinelloides*, Sp = *Schizosaccharomyces pombe*, Cp = *Cryphonectria parasitica,* Ce = *Caenorhabditis elegans*, At = *Arabidopsis thaliana*, Dm = *Drosophila melanogaster*. *Rhizophagus irregularis* proteins are identified by JGI numeric codes. *D. melanogaster, A. tahliana* and *C. elegans* proteins are identified (after the two-letter prefix) by FunRNA (Choi et al. 2014) ID. Protein ID of other species (NCBI or JGI): MoAGO1 = XP_003716704.1, MoAGO2 = XP_003717504.1, MoAGO3 = XP_003714217.1, NcQDE-2 = XP_011394903.1, NcSMS-2 = EAA29350.1, SpAGO1 = O74957.1, McAGO-1 = 104161, McAGO-2 = 195366, McAGO-3 = 104163, CpAGL1 = ACY36939.1, CpAGL2 = ACY36940.1, CpAGL3 = ACY36941.1, CpAGL4 = ACY36942.1. The numbers at the nodes are bootstrap values (%) for 1000 replications. Figure was generated with Evolview v2.

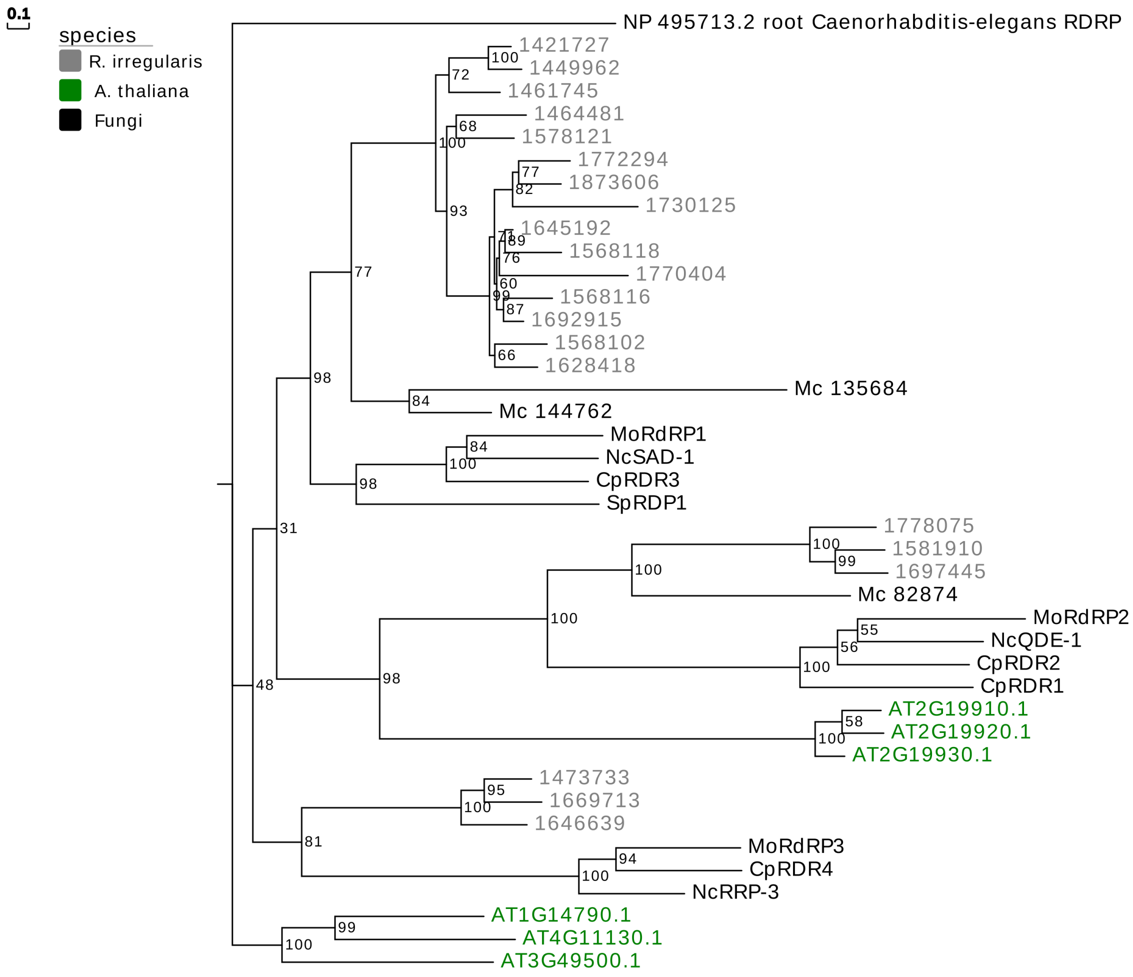

Figure S2. Phylogenetic relationship of RdRp proteins in different organisms. Proteins are discernible by species according to color and a two-letter prefix: Mo = *Magnaporthe oryzae*,Nc = *Neurospora crassa*,Mc = *Mucor circinelloides*, Sp = *Schizosaccharomyces pombe*, Cp = *Cryphonectria parasitica*, At = *Arabidopsis thaliana*. *Rhizophagus irregularis* proteins are identified by JGI numeric codes. *A. thaliana* proteins are identified (after the two-letter prefix) by FunRNA (Choi et al. 2014) ID. Protein ID of other species (NCBI or JGI): MoRdRP1= XP_003721007.1, MoRdRP2 = XP_003711624.1, MoRdRP3 = XP_003712093.1, NcQDE-1 = EAA29811.1, NcSAD-1 = XP_964248.3, NcRRP-3 = XP_963405.1, SpRDP1 = NP_001342838.1, McRdRP-1 = 111871, McRdRP-2 = 104159, CpRDR1 = 270014, CpRDR2 =35624, CpRDR3 = 10929, CpRDR4= 339656. The numbers at the nodes are bootstrap values (%) for 1000 replications. Tree was rooted using *Arabidopsis thaliana* Argonaute 6 (NCBI Reference Sequence: NP_180853.2). Figure was generated with Evolview v2.

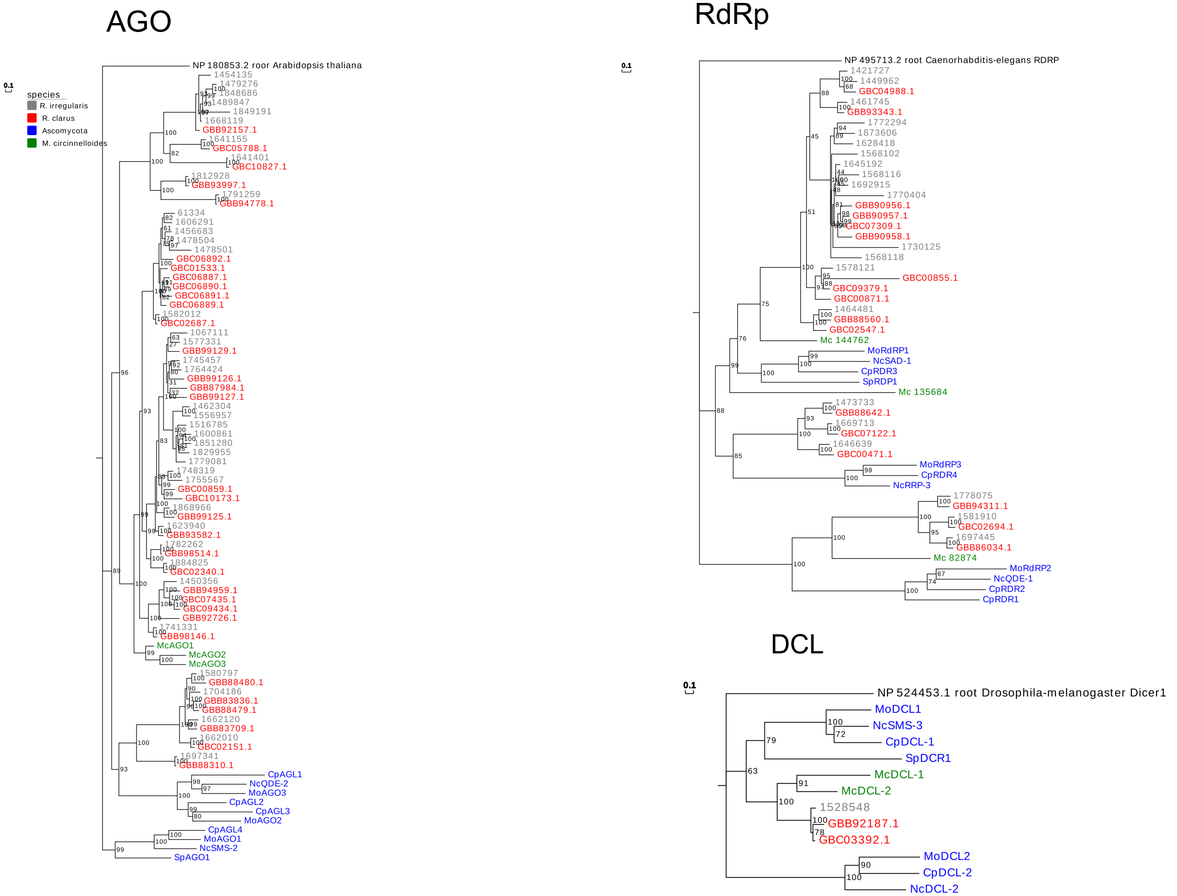

Figure S3. Phylogenetic relationship of AGO, RdRp and DCL proteins in different fungi. Proteins are discernible by species according to color and a two-letter prefix: Mo = *Magnaporthe oryzae*,Nc = *Neurospora crassa*, Mc = *Mucor circinelloides*, Sp = *Schizosaccharomyces pombe*, Cp = *Cryphonectria parasitica*. *Rhizophagus irregularis* proteins are identified by JGI numeric codes. *Rhizophagus clarus* proteins are identified with NCBI protein ID. Protein ID of other species (NCBI or JGI): MoAGO1 = XP_003716704.1, MoAGO2 = XP_003717504.1, MoAGO3 = XP_003714217.1, NcQDE-2 = XP_011394903.1, NcSMS-2 = EAA29350.1, SpAGO1 = O74957.1, McAGO-1 = 104161, McAGO-2 = 195366, McAGO-3 = 104163, CpAGL1 = ACY36939.1, CpAGL2 = ACY36940.1, CpAGL3 = ACY36941.1, CpAGL4 = ACY36942.1, MoRdRP1= XP_003721007.1, MoRdRP2 = XP_003711624.1, MoRdRP3 = XP_003712093.1, NcQDE-1 = EAA29811.1, NcSAD-1 = XP_964248.3, NcRRP-3 = XP_963405.1, SpRDP1 = NP_001342838.1, McRdRP-1 = 111871, McRdRP-2 = 104159, CpRDR1 = 270014, CpRDR2 =35624, CpRDR3 = 10929, CpRDR4= 339656, MoMDL1 = XP_003714515.1, MoMDL2 = XP_003715365.1, NcSMS-3 = XP_961898.1, NcDCL-2 = XP_963538.3, SpDCR1 = NP_588215.2, McDCL-1 = CAK32533.1, McDCL-2 = CAZ65730.1, CpDCL-1 = ABB00356.1, CpDCL-2 = ABB00357.1. Trees were rooted using: *Arabidopsis thaliana* Argonaute 6 (NCBI Reference Sequence: NP_180853.2) for AGO, *Caenorhabditis elegans* RdRP (NCBI Reference Sequence: NP_495713.2) for RdRp and *Drosophila melanogster* Dicer 1 (NCBI Reference Sequence: NP_524453.1) fro DCLThe numbers at the nodes are bootstrap values (%) for 1000 replications. Figure was generated with Evolview v2.

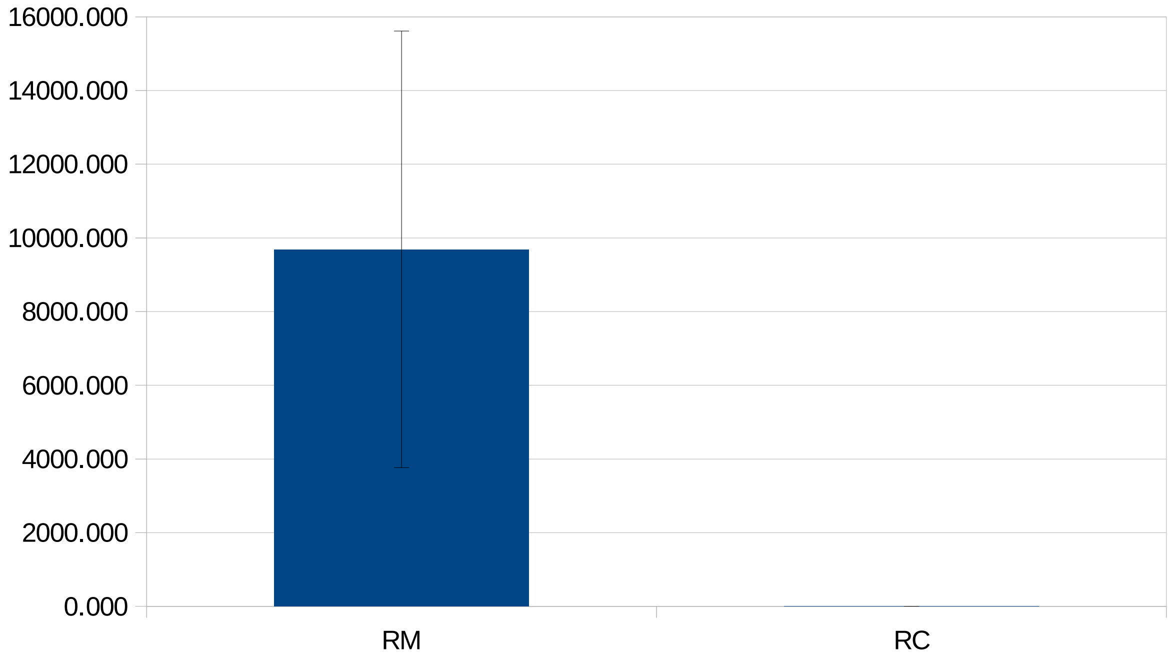

Figure S4. Expression of *MtPT4* relative to *MtTEF* assessed by qRT-PCR in RM samples (mycorrhizal roots) compared to RC ones (non-mycorrhizal roots). Data for each condition are presented as mean ± standard error.

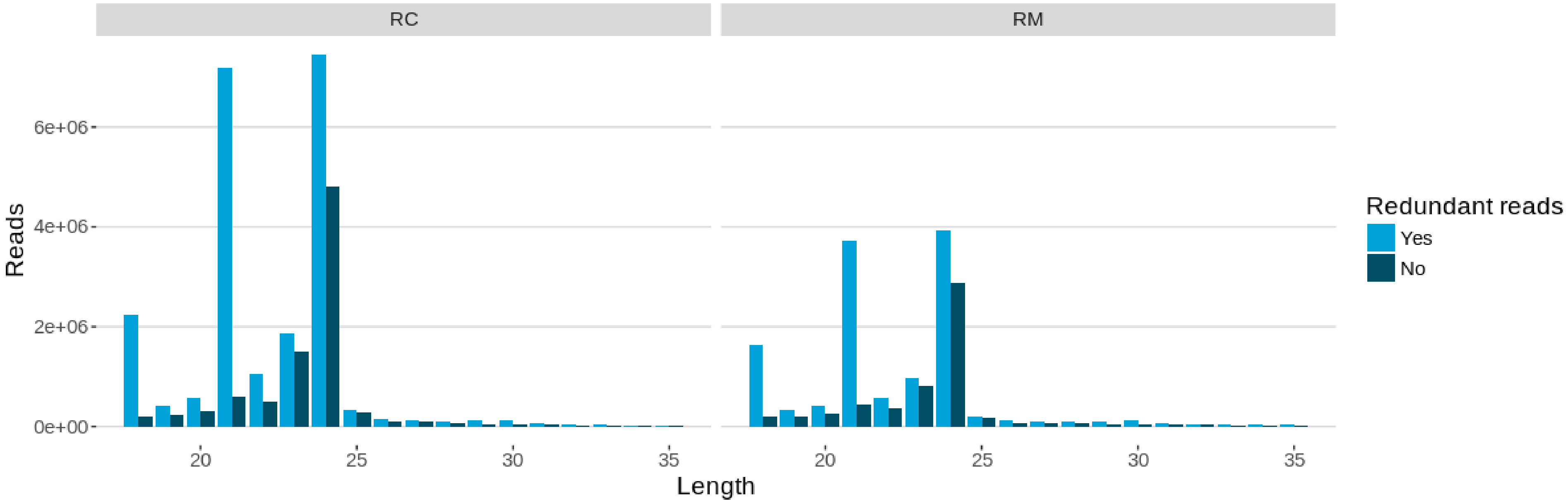

Figure S5. Length distribution (expressed in nucleotide) of sRNAs reads (redundant and non-redundant) from RC (non mycorrhizal roots) and RM (mycorrhizal roots) libraries mapping on *Medicago truncatula* genome.

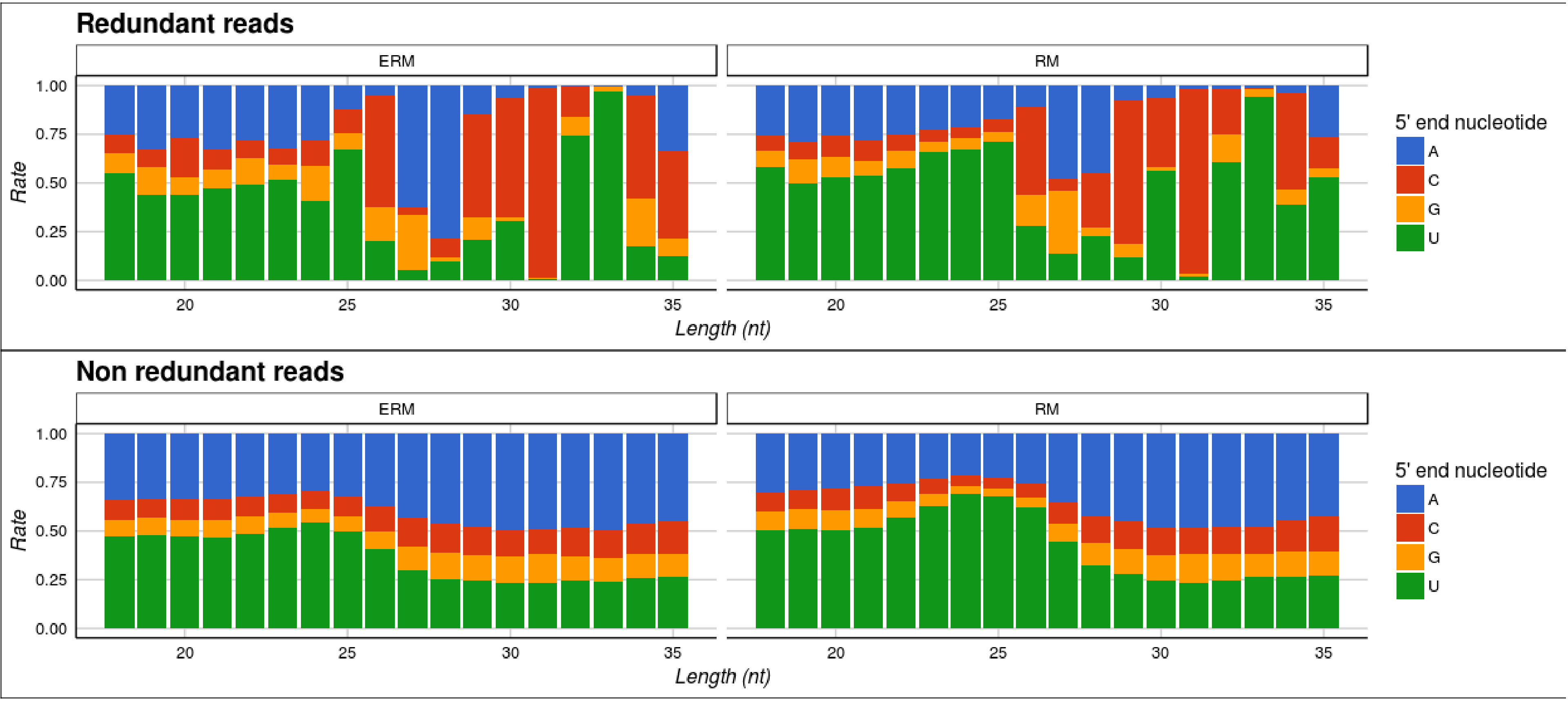

Figure S6. Relative nucleotide frequency of 5' end of sRNAs reads (redundant and non-redundant) from RM (mycorrhizal roots) and ERM (extra radical mycelium) libraries mapping on *Rhizophagus irregularis* genome.

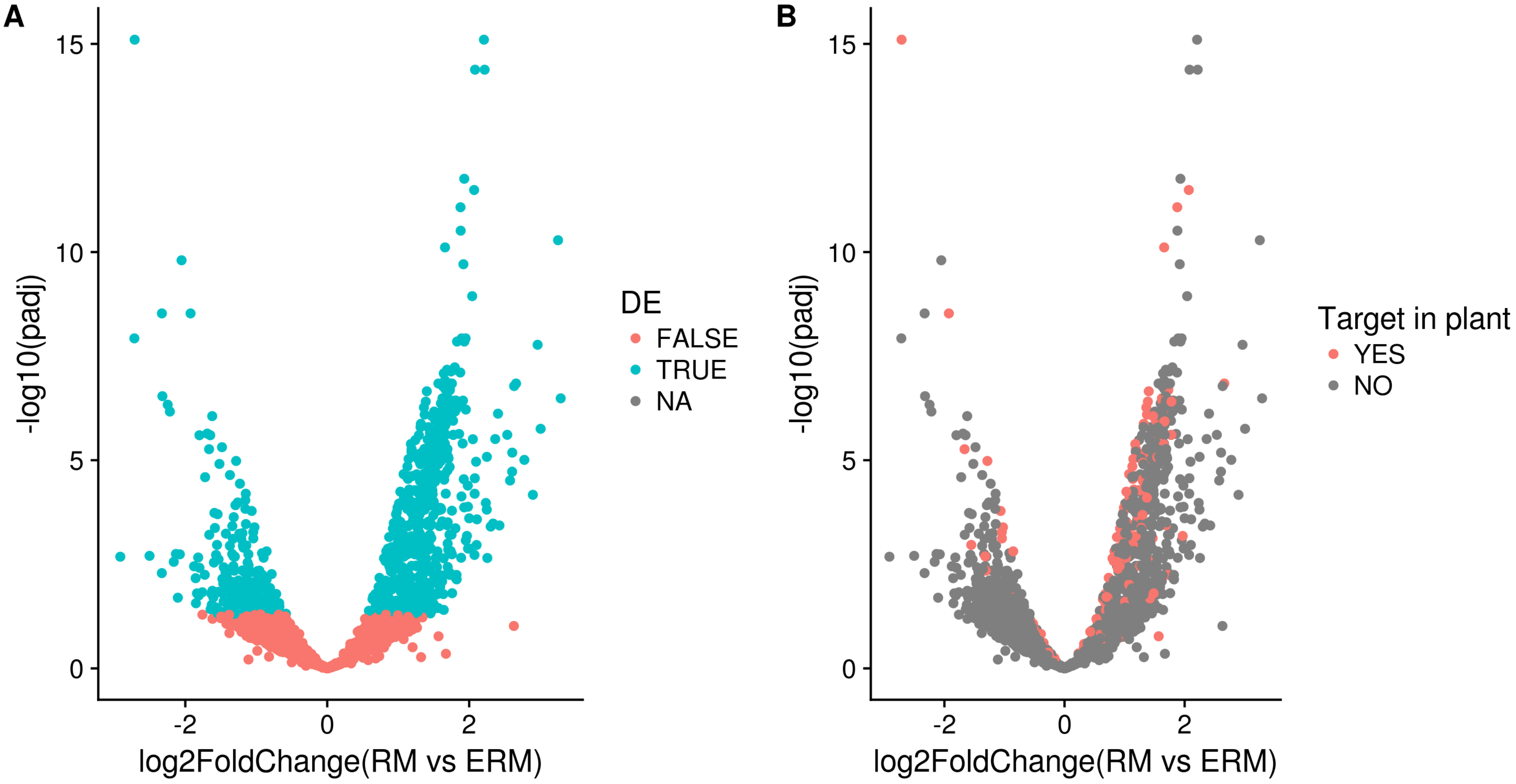

Figure S7. Volcano plots (fold changes vs adjusted p-values) of *Rir*-sRNA-generating loci. **A)** Blue dots (TRUE) represent the loci differentilally expressed (adjusted p-values < 0.05) between between ERM (extra radical mycelium) and RM (mycorrhizal root) conditions according to DESeq2 analysis (DE = differentially expressed); **B)** Red dots (YES) represent the loci that produce *Rir*-sRNAs targeting *Mtr*-mRNAs, according to sPARTA analysis.

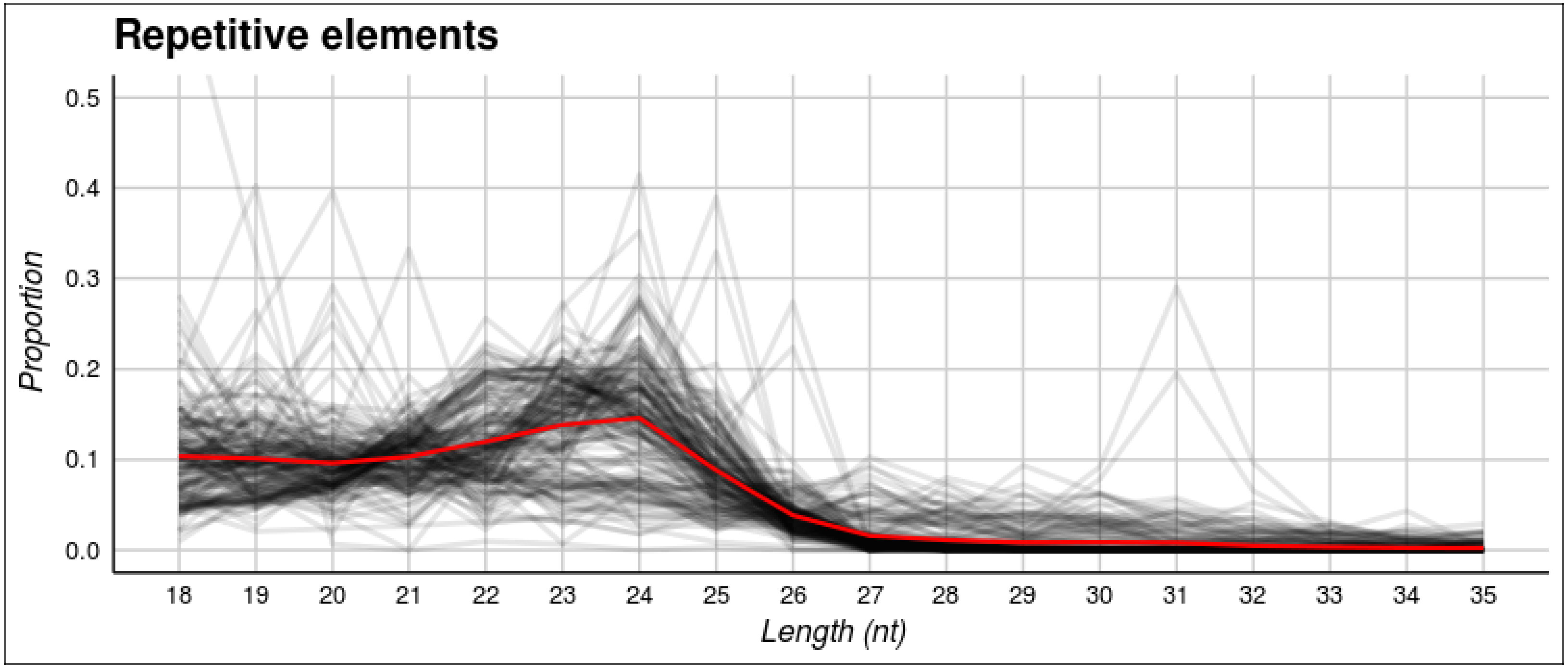

Figure S8. Length distribution (in nucleotide) of sRNA reads that defined the *Rir*-sRNAs-generating loci homologous to repetitive elements in RepBase. Black lines are the length distribution of the individual loci and red line is the average length distribution of the plotted loci.
